# Tumor-immune crosstalk in lung cancer: emerging roles of long non-coding RNAs

**DOI:** 10.3389/fimmu.2026.1832599

**Published:** 2026-05-29

**Authors:** Upasna Madan, Riitta Lahesmaa, Anil K. Thotakura

**Affiliations:** 1 Turku Bioscience Center, University of Turku and Åbo Akademi University, Turku, Finland; 2 InFLAMES Research Flagship Center, University of Turku, Turku, Finland; 3 Immuno-Oncology, Oncology Research, Orion Pharma, Turku, Finland; 4 Institute of Biomedicine, University of Turku, Turku, Finland

**Keywords:** biomarker, long non coding RNA, NSCLC, PD-L1, tumor immune microenvironment

## Abstract

Lung cancer, primarily non-small cell lung cancer (NSCLC), remains one of the leading contributors to cancer-related mortality worldwide. The tumor immune microenvironment (TIME) critically influences tumor progression, metastasis, prognosis, and therapeutic responses. Emerging evidence highlights the significant role of long non-coding RNAs (lncRNAs) in mediating tumor-immune interactions, thereby underscoring their potential as biomarkers and therapeutic targets. This review synthesizes current knowledge of lncRNAs expressed by immune and tumor cells in NSCLC. Immune cell-derived lncRNAs regulate the differentiation and function of specific immune cell subsets; tumor cell-derived lncRNAs modulate immune checkpoint molecules, immune evasion pathways, and immune cell infiltration and polarization. Collectively, these lncRNAs shape anti-tumor immunity and therapy responses. We provide an overview of their expression, prognostic relevance, functional effects, and underlying molecular mechanisms, classified by level of evidence spanning clinical, preclinical, and in silico studies. We then discuss convergent regulatory nodes shared across lncRNAs, lncRNA-mediated immune checkpoint inhibitor response and resistance, and therapeutic targeting strategies. Finally, we highlight emerging technological frontiers for lncRNA profiling in the TIME, alongside key limitations and future directions of the field.

## 1 Introduction

Lung cancer is one of the most prevalent and lethal malignancies worldwide, being the leading cause of cancer-related deaths across various populations. According to the 2022 global cancer statistics, lung cancer accounts for approximately 12.4% of all new cancers and holds a mortality rate of 18.7%, illustrating its significant impact on global health (1). The two primary histological classifications of lung cancer are non-small cell lung cancer (NSCLC), which accounts for approximately 80–85% of cases, and small cell lung carcinoma (SCLC), comprising the remaining 15-20% (2, 3). Among NSCLC subtypes, adenocarcinoma (AD) and squamous cell carcinoma (SCC) are the most prevalent, accounting for approximately 50% and 40% of cases, respectively (3).

Despite major strides in the diagnosis and treatment of lung cancer, survival outcomes—especially for patients with advanced-stage disease—remain suboptimal (4, 5). Early-stage NSCLC patients have the best prognosis. Unfortunately, the majority of patients—around 75%—are diagnosed at a later stage (III or IV), when curative treatment is less effective (4). Over the past four decades, the disease’s severity has been evident in its consistently low 5-year relative survival rate, which has remained below 21% despite gradual improvements (5). These figures highlight the persistent need for early detection and more effective treatment strategies.

The tumor microenvironment (TME) is a complex and dynamic ecosystem that plays a critical role in the growth, progression, and treatment response of lung cancer (6, 7). In addition to cancerous cells, the TME comprises diverse cellular components such as immune cells, fibroblasts, and endothelial cells, and is enriched with various signaling molecules—including growth factors, proangiogenic mediators, cytokines, and chemokines—as well as structural elements of the extracellular matrix (6, 7).

The tumor immune microenvironment (TIME) comprises various immune cell types, predominantly tumor-infiltrating lymphocytes (TILs) and myeloid-derived populations (6–8). Immune cells can either suppress or promote tumor progression depending on their polarization and functional state (7, 8). For instance, Tumor-associated macrophages (TAMs) often adopt an immunosuppressive phenotype that facilitates tumor survival and progression, while CD8+ T cells are generally associated with anti-tumor immunity (7, 8). Recent single-cell RNA sequencing (scRNA-seq) studies have provided deeper insights, showing immune remodeling during lung adenocarcinoma progression, including expansion of monocyte-derived macrophages, plasmacytoid dendritic cells, and regulatory T cells at metastatic sites, accompanied by exhaustion of CD8^+^ T cells and natural killer cells (9). The balance of these immune cell populations significantly influences patient outcomes, underscoring the importance of immune profiling in clinical settings (10, 11). A meta-analysis of >15000 NSCLC patients further confirmed that increased CD8^+^, CD3^+^, and CD4^+^ TIL densities correlate with improved overall survival, whereas increased Foxp3 TIL densities were associated with poor overall survival (12). Immune checkpoint Inhibitors (ICIs) have further underscored the clinical importance of TIME in NSCLC. Antibodies targeting programmed cell death 1 (PD-1), in combination with cytotoxic T-lymphocyte-associated protein 4 (CTLA-4) inhibition, demonstrate durable responses in patients with NSCLC as first-line treatment (13, 14). However, only a subset of patients benefits, and response is influenced by tumor-intrinsic and immune factors, including the presence and functional state of TILs and tumor mutational burden (15–17). This variability suggests that additional regulatory layers shape antitumor immunity and therapy response. Emerging evidence indicates that long non-coding RNAs (lncRNAs) are important regulators of these pathways.

**Figure 1:**
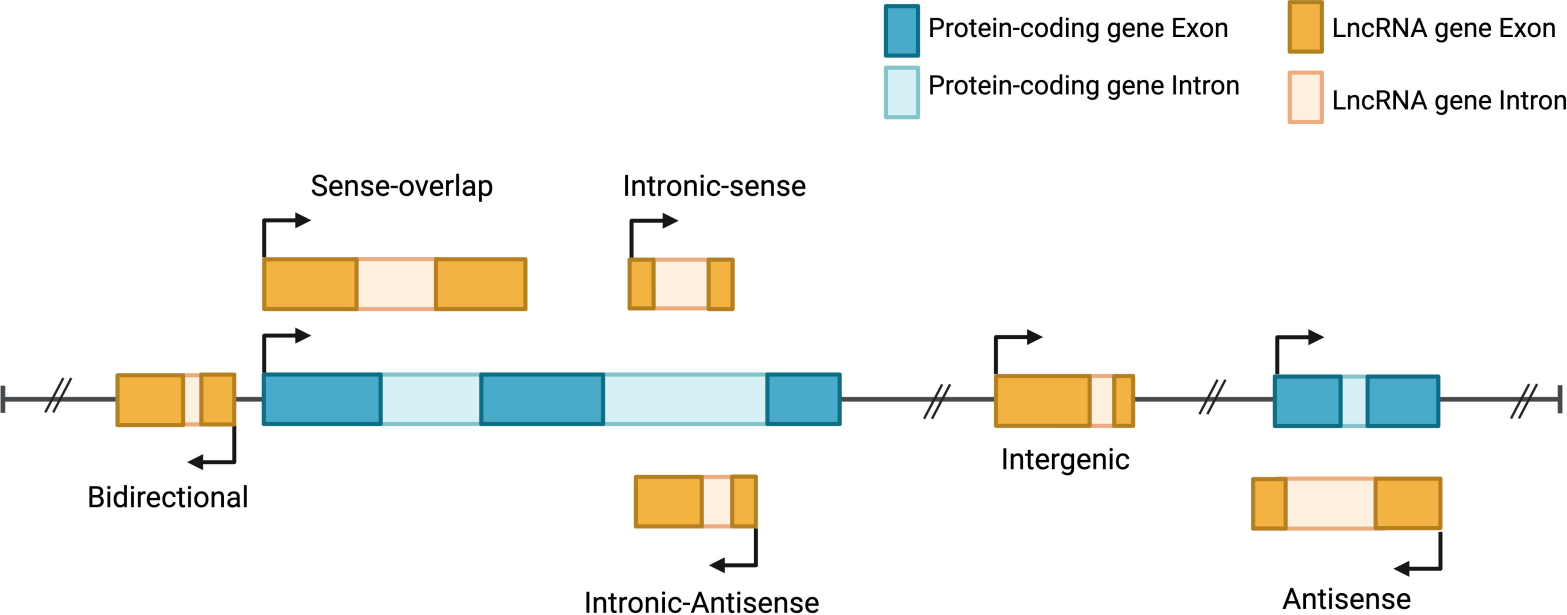
Classification of long non-coding RNAs (lncRNAs) based on their genomic proximity to protein-coding genes. LncRNAs are categorized according to their genomic location and transcriptional orientation relative to nearby protein-coding genes. Sense lncRNAs are transcribed from the same strand as a protein-coding gene and either overlap one or more of its exons (sense-overlap) or lie entirely within an intron (intronic-sense). Antisense lncRNAs are transcribed from the opposite strand of a protein-coding gene, either overlapping its exons in reverse orientation or lying entirely within an intron (intronic-antisense). Bidirectional lncRNAs are transcribed from the opposite strand within ~1 kb upstream of a protein-coding gene’s promoter. Intergenic lncRNAs (lincRNAs) are located in genomic intervals between two protein-coding genes, with no overlap. Filled boxes represent exons; lighter intervening regions represent introns. Bent arrows indicate transcription start sites and direction. Double-slash marks (//) denote genomic distance not drawn to scale. Yellow, lncRNA gene; blue, protein-coding gene. Created with BioRender.com.

LncRNAs are crucial players in the genomic landscape, challenging the traditional view that only protein-coding genes are significant in biological processes. Although nearly 75% of the human genome is transcribed, only about 2% encodes proteins, leaving the majority as non-coding transcripts (18). Among these, lncRNAs constitute a major, functionally diverse subgroup. They are defined as transcripts longer than 200 nucleotides lacking protein-coding potential (19, 20). They are typically generated by RNA polymerase II and are spliced and polyadenylated, leading to their description as “mRNA-like” (21). Based on their genomic proximity to protein-coding genes, they are classified into five types: sense, antisense, bidirectional, intronic, and intergenic (**Figure 1**) (22).

**Figure 2:**
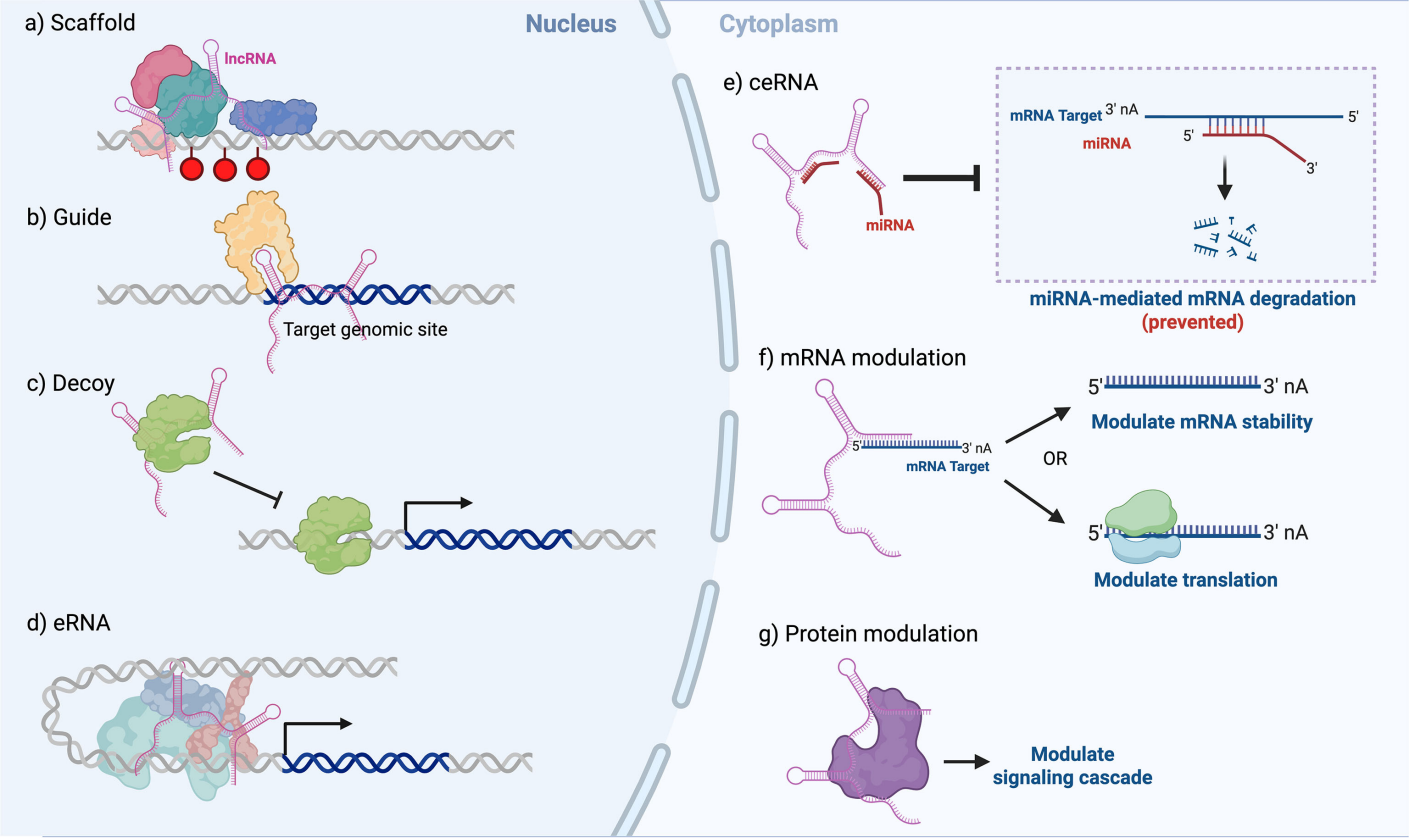
Molecular mechanisms of Long non-coding RNAs (lncRNAs). LncRNAs operate through diverse mechanisms in the nucleus (panels **(A–D)**) and cytoplasm (panels **(E–G)**). **(A)** Scaffold: lncRNAs serve as structural platforms that bring multiple regulatory proteins into a single complex, for example by recruiting chromatin-modifying complexes. **(B)** Guide: lncRNAs direct individual transcriptional regulators to sequence-specific target genomic sites. **(C)** Decoy: lncRNAs sequester transcriptional activators or repressors away from their target genes, acting as molecular sponges. **(D)** Enhancer RNAs (eRNAs): lncRNAs facilitate chromatin looping between enhancers and promoters to promote transcriptional activation. **(E)** *Competitive endogenous RNAs (ceRNAs*): lncRNAs sequester miRNAs through complementary base-pairing, preventing miRNA-mediated decay or translational repression of target mRNAs. **(F)** mRNA modulation: lncRNAs interact with *mRNAs*, modulating their stability, degradation, or to regulate translation efficiency by influencing ribosome recruitment. **(G)** Protein modulation: lncRNAs bind to cytoplasmic proteins, affecting their stability, localization, or activity, and thereby fine-tuning intracellular signaling cascades. Red dots represent modification marks deposited on chromatin; bent arrows indicate transcriptional activation; T-bars indicate inhibition or sequestration. Created with BioRender.com.

Mechanistically, they interact with DNA, RNA, and proteins, thereby regulating transcription, post-transcriptional processing, and epigenetic modifications (22). They can function as molecular decoys, scaffolds, guides, or sponges for their binding partners (**Figure 2**) (22). In the nucleus, they regulate gene expression in *cis* or *trans*, modulate alternative splicing, maintain genome stability, and contribute to nuclear organization (23–27). In the cytoplasm, they interact with signaling proteins, regulate mRNA stability and translation, and act as competitive endogenous RNAs (ceRNA) by sponging microRNAs (28, 29).

LncRNAs are expressed in a cell type-specific and context-dependent manner, with their expression varying across developmental stages and in response to environmental cues (30). They are typically present at low copy numbers owing to their regulatory nature (30). They exhibit differential expression in various disease states, including cancer (31, 32). In the TME, lncRNAs are increasingly implicated in modulating processes such as cell proliferation, apoptosis, angiogenesis, epithelial-to-mesenchymal transition (EMT), immune evasion, and therapeutic response (33–36). In lung cancer, particularly AD and SCC, lncRNAs contribute to distinct immune regulatory patterns; immune suppression in AD is largely driven by T cell exclusion, whereas SCC is characterized by T cell dysfunction, with subtype-specific lncRNA signatures predicting patient prognosis and immunotherapy response (37). A growing body of work has identified lncRNAs with cell type-specific expression and function in the lung TIME, including those operating within tumor cells, TAMs, myeloid-derived suppressor cells (MDSCs), and CD8^+^ T cells. Despite this progress, their expression and function in CD4^+^ T cell subsets — particularly regulatory T cells (Tregs) — dendritic cells (DCs), and natural killer (NK) cells remain largely unexplored. Given that these populations are also central determinants of immune evasion and immunotherapy response, this imbalance represents a significant gap that constrains our mechanistic understanding of TIME regulation in lung cancer.

In this review, we synthesize the current understanding of lncRNAs expressed by immune and tumor cells in lung cancer, focusing on their roles in shaping the tumor immune microenvironment and influencing disease progression. For each lncRNA, we summarize its expression, prognostic associations, functional effects, and underlying mechanisms, together with the level of evidence supporting these findings — categorized as clinical (evidence from human patient samples), preclinical (cell line and animal model studies), in silico (computational analysis of public datasets), or a combination thereof. We then discuss emerging themes and convergent regulatory nodes, followed by the clinical implications of lncRNAs as biomarkers, their roles in ICI response and resistance, and current therapeutic targeting strategies. Finally, we outline the technological frontiers enabling lncRNA discovery in the TIME and the limitations and future directions of the field. While recent reviews have addressed lncRNAs in the lung cancer TME (38, 39) and in the NSCLC TIME specifically (40), the present review differs in scope and emphasis. We organize immune-regulatory lncRNAs by the cell type in which they act, and we classify each lncRNA by level of evidence as described above, enabling readers to distinguish high-confidence translational candidates from hypothesis-generating observations. We also incorporate the most recent advances, including lncRNA-mediated immunotherapy resistance and emerging technologies that are reshaping how lncRNAs can be studied in the TIME at single-cell and spatial resolution.

## 2 Immune cell-derived lncRNAs regulating the lung tumor immune microenvironment

**Figure 3:**
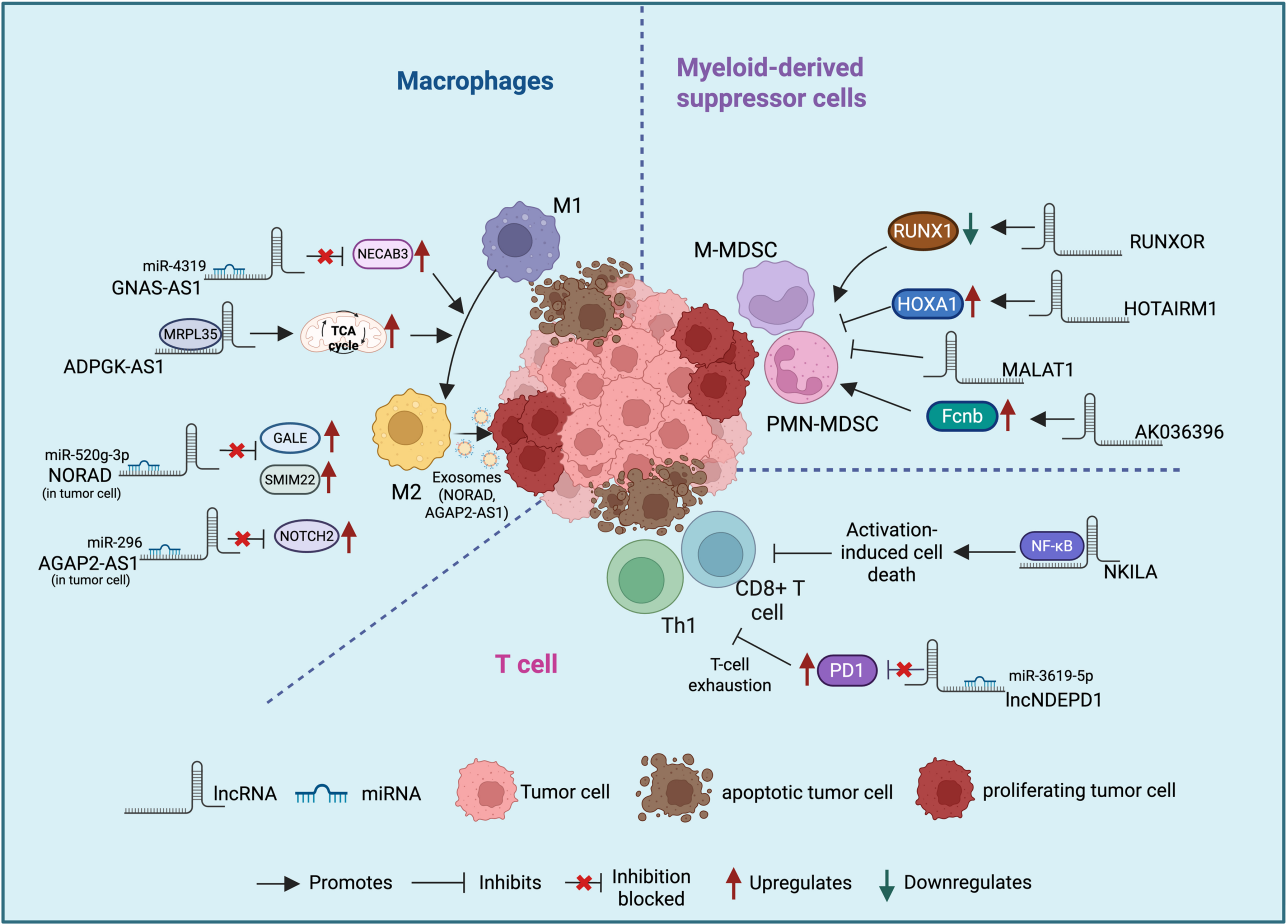
Immune-cell derived lncRNAs regulating the lung tumor immune microenvironment. *GNAS-AS1* and *ADPGK-AS1* drive macrophage polarization from the pro-inflammatory M1 to the immunosuppressive M2 phenotype, thereby promoting tumor proliferation. *AGAP2-AS1* and *NORAD* are secreted via M2 macrophage–derived exosomes and enhance tumor cell proliferation. *RUNXOR* facilitates the accumulation and immunosuppressive functions of myeloid-derived suppressor cells (MDSCs), whereas *HOTAIRM1* and *MALAT1* inhibit MDSC-mediated suppression. AK036396 specifically supports PMN-MDSC immunosuppressive function (in murine models). In T cells, *NKILA* promotes activation-induced cell death (AICD) of cytotoxic T lymphocytes (CTLs) and Th1 cells by inhibiting NF-κB signaling, while *lncNDEPD1* contributes to T-cell exhaustion through PD-1 upregulation. Created with BioRender.com.

Immune cell-derived lncRNAs play pivotal roles in regulating the functional states of immune cells and shaping their interactions with tumor cells and the surrounding microenvironment. For instance, lncRNAs can modulate macrophage polarization toward a pro-tumorigenic phenotype or influence the differentiation and activity of T cells, which are critical for orchestrating anti-tumor immunity. Through such mechanisms, immune cell-derived lncRNAs emerge as key regulators of the TME, with potential applications as biomarkers and therapeutic targets. While significant progress has been made in understanding their broad influence on tumor–immune dynamics, a more precise characterization of lncRNAs expressed in specific immune subsets remains necessary. The following subsections discuss these subset-specific lncRNAs in detail, highlighting how they reprogram critical immune populations within the lung cancer TME (**Table 1****;** **Figure 3**).

**Table 1:** Summary of subset-specific lncRNAs expressed by immune cells in the NSCLC tumor microenvironment.

LncRNA	Cell Type	Status in cancer	Prognosis	Effects	Mechanism	Functional validation	Level of evidence	Reference
GNAS-AS1	TAMs, NSCLC tumor cells	Upregulated	Poor prognosis (high GNAS-AS1 expression, lower OS and MFS)	Promotes macrophage M2 polarization; tumor cell migration, invasion, and proliferation	Sponges miR-4319 leading to upregulation of NECAB3	*In vitro* knockdown and overexpression assays	Clinical + Preclinical	(44)
NORAD	M2 macrophages, M2-derived Evs, NSCLC tumor cells	Upregulated	Not reported	Enhances glycolysis, cell proliferation, and tumor growth	Sponges miR-520g-3p → upregulates SMIM22 → activates GALE	EV isolation, *in vitro* assays, *in vivo* –mouse xenograft tumor models	Clinical + Preclinical	(45)
ADPGK-AS1	TAMs	Upregulated	Poor prognosis (High MRPL35 (ADPGK-AS1 target) linked to low DFS and OS)	Promotes M2-like polarization, enhances tumor-promoting cytokine release, suppresses anti-tumor immunity	Binds mitochondrial ribosomal proteins (MRPL35 and MRPL15), enhances TCA cycle and mitochondrial fission to drive M2 polarization	*In vitro* assays, ex vivo (lung slices), and *in vivo* -mouse xenograft tumor model	Clinical + Preclinical	(47)
AGAP2-AS1	M2 macrophages, M2-derived exosomes, NSCLC tumor cells	Upregulated	Poor prognosis (high AGAP2-AS1 expression lower OS and DFS)	Enhances tumor cell proliferation, invasiveness, radioresistance; reduces NK cell cytotoxicity	Sponges miR-296 to upregulate NOTCH2	*In vitro* assays; *in vivo* -mouse xenograft tumor model	Clinical + Preclinical	(48)
RUNXOR	MDSCs	Upregulated	Not reported	Promotes MDSCs	Suppresses RUNX1, promotes Arg1 expression in MDSCs	*In vitro*- siRNA-mediated knockdown	Clinical + Preclinical	(53)
HOTAIRM1	MDSCs	Downregulated	Not reported	Downregulation of HOTAIRM1 increases MDSC-mediated immunosuppression, inhibits granulocytes differentiation; inhibits Th1/CTL responses	Upregulates HOXA1, reduces Arg1 and suppressive molecules	*In vitro* overexpression assay; *in-vivo* (HOXA1 overexpression in mouse tumor model; no direct HOTAIRM1 modulation)	Clinical + Preclinical	(55)
MALAT1	PBMCs	Downregulated	Not reported	Downregulation of MALAT1 increases MDSCs, reduces CD8^+^ T cells	Unknown	*In vitro*- siRNA-mediated MALAT1 knockdown in PBMCs increased MDSC frequency	Clinical + Preclinical	(56)
AK036396	PMN-MDSCs (murine)	Not reported	Not reported	Promotes PMN-MDSCs immunosuppressive function, impairs MDSC maturation	Stabilizes Ficolin B (Fcnb)	*In vitro* siRNA mediated knockdown, *in-vivo* mouse lung tumor model	Preclinical	(57)
NKILA	CTLs and Th1 cells	Upregulated	Poor prognosis (high NKILA expression in CTLs linked to poorer DFS)	Promotes AICD of CTLs and Th1 cells leading to immune evasion	Calcium signaling induces STAT1-mediated NKILA transcription, which inhibits NF-κB	*In vitro* -shRNA knockdown, *in vivo*-mouse xenograft tumor model	Clinical + Preclinical	(66)
lncNDEPD1	CD8^+^ T cells	Upregulated	Not reported	Increases PD-1 expression; contributes to T cell exhaustion	Sponges miR-3619-5p, stabilizes PDCD1 mRNA upregulating PD-1	*In vitro*- si RNA mediated knockdown; *in vivo* -knockdown in CAR-T cells enhanced their cytotoxicity in xenograft tumor model	Clinical + Preclinical	(67)

The table includes the cell type in which lncRNAs are expressed, expression status in cancer, associated prognosis, functional effects on tumor progression or immune modulation, molecular mechanisms, validation approaches, level of supporting evidence, and references.

Level of Evidence: *Clinical* — evidence from human patient samples; *Preclinical* — functional characterization in cell lines or animal models; *In silico* — computational analysis of public datasets (e.g., TCGA, GEO) without accompanying wet-lab validation. Studies combining multiple approaches are labelled accordingly (e.g., *Clinical + Preclinical*).

OS, overall survival; MFS, metastasis-free survival; DFS, disease-free survival; EVs, extracellular vesicles; MDSCs, myeloid-derived suppressor cells; PMN-MDSCs, polymorphonuclear myeloid-derived suppressor cells; CTLs, cytotoxic T lymphocytes; Th1, T helper 1 cells; TAMs, tumor-associated macrophages; PBMCs, peripheral blood mononuclear cells; AICD, activation-induced cell death; NK cells, natural killer cells.

### 2.1 Tumor-associated macrophages

Macrophages that accumulate within the TME, commonly referred to as TAMs, play a pivotal role in shaping immune responses and influencing tumor progression (41, 42). Depending on microenvironmental cues and their activation status, macrophages can be broadly classified into two different functional states: the pro-inflammatory M1 and the anti-inflammatory M2 (41, 42). *In vitro*, M1-like macrophages are induced by lipopolysaccharide (LPS) and/or interferon-γ (IFN-γ) and are characterized by high expression of pro-inflammatory cytokines such as TNF-α, IL-1β, IL-6, IL-12, and IL-23, along with production of nitric oxide (NO) and reactive oxygen species (ROS). In contrast, M2-like macrophages, induced by IL-4, express high levels of anti-inflammatory cytokines IL-10 and TGF-β, as well as arginase, an enzyme involved in immune regulation (41, 42). M1-like TAMs are associated with suppressing tumor growth, inhibiting migration, and inhibiting angiogenesis, while M2-like TAMs exhibit anti-inflammatory properties and support tumor progression, invasion, angiogenesis, and metastasis (41, 42). Additionally, TAMs are predominantly skewed toward the M2-like phenotype and are often linked to poor prognosis (41, 42). In NSCLC patients, elevated serum levels of TAMs have shown a strong correlation with disease progression and may serve as a sensitive and specific biomarker for monitoring chemotherapy response (43).

Emerging evidence suggests that lncRNAs contribute to the functional reprogramming of TAMs, thereby amplifying their pro-tumorigenic roles within the lung cancer TME. **GNAS-AS1** is one of the best-characterized examples. It is highly expressed in TAMs, clinical tumor tissues, and NSCLC cell lines, and elevated levels correlate with poor overall survival (OS) and metastasis-free survival (MFS) (41). Functionally, GNAS-AS1 promotes NSCLC cell proliferation, migration, and invasion, while driving macrophage polarization toward the immunosuppressive M2 phenotype. Mechanistically, it acts as a ceRNA, sponging miR-4319 and thereby relieving repression of its target, NECAB3, a calcium-binding protein that facilitates M2 polarization and tumor progression (44).

Another pro-tumorigenic lncRNA, **NORAD,** is secreted by M2-polarized TAMs through extracellular vesicles (EVs) (45). In recipient tumor cells, NORAD functions as a ceRNA for miR-520g-3p, thereby upregulating SMIM22. SMIM22, a small membrane-associated protein with emerging links to cancer development (46), in turn enhances GALE (UDP-galactose 4-epimerase) expression, which supports glycolysis and proliferation in NSCLC cells. Functional assays using A549 cells and xenograft mouse models showed that M2 macrophage-derived EVs enriched in NORAD markedly enhanced tumor proliferation and growth. Consistently, NSCLC tissues display higher levels of NORAD and SMIM22 and reduced miR-520g-3p compared with adjacent non-tumor tissues (45).

**ADPGK-AS1**, a mitochondria-associated lncRNA, represents a key immunometabolic regulator of TAMs. It is significantly upregulated in TAMs from lung cancer patients as well as in M2-like macrophages and cancer-conditioned macrophages *in vitro*. ADPGK-AS1 localizes partly to mitochondria, where it binds MRPL35 and MRPL15—particularly MRPL35, whose high expression correlates with poor disease-free survival (DFS) and OS in NSCLC patients. ADPGK-AS1 enhances tricarboxylic acid (TCA) cycle activity and mitochondrial fission, thereby promoting M2 polarization, immunosuppressive cytokine release, and tumor progression. Conversely, macrophage-specific silencing of ADPGK-AS1 induces an M1-like phenotype characterized by elevated pro-inflammatory cytokines and ROS, thereby suppressing tumor growth in co-cultures, ex vivo lung tumor slices, and *in vivo* models (47).

AGAP2-AS1 has been linked to therapy resistance. It is upregulated in radioresistant NSCLC tissues and is enriched in exosomes derived from M2-polarized macrophages. Mechanistically, AGAP2-AS1 sponges miR-296, thereby derepressing NOTCH2, a transcriptional regulator associated with tumor survival and radiotherapy resistance. Clinically, patients with high AGAP2-AS1 or NOTCH2 expression, or low miR-296 levels, exhibit worse OS and DFS. Functionally, exosomal AGAP2-AS1 is taken up by tumor cells, enhancing their proliferation, invasiveness, resistance to radiotherapy, and resistance to NK cell-mediated cytotoxicity. Targeting AGAP2-AS1 or restoring miR-296 expression sensitizes NSCLC cells to radiation and NK cell-mediated cytotoxicity (48).

Collectively, these studies underscore the diverse mechanisms by which TAM-derived lncRNAs—via ceRNA networks, metabolic rewiring, and intercellular communication—reprogram macrophages and tumor cells alike, thereby fostering an immunosuppressive and therapy-resistant microenvironment.

### 2.2 Myeloid-derived suppressor cells

MDSCs are a heterogeneous population of immature myeloid cells that differentiate into mature cells such as macrophages, granulocytes, and dendritic cells under normal physiological conditions (49, 50). However, in pathological conditions such as cancer, their normal differentiation pathway is disrupted, and these cells remain arrested in an immature yet activated and highly suppressive state (49, 50). In humans, MDSCs are phenotypically defined by the coexpression of CD11b and CD33, along with the absence of HLA-DR, a major histocompatibility complex class II molecule (CD11b^+^CD33^+^HLA-DR^-^) (51). Based on their surface marker profiles, MDSCs are classified into two main subsets. Polymorphonuclear MDSCs (PMN-MDSCs; also referred to as granulocytic MDSCs or G-MDSCs) are characterized as CD11b^+^CD33^+^HLA-DR^-^CD15^+^CD14^-^, while monocytic MDSCs (M-MDSCs) exhibit a CD11b^+^CD33^+^HLA-DR^low/^-^CD14^+^ phenotype (51). In cancer, MDSCs exhibit potent immunosuppressive and tumor-promoting activities (52). These include the secretion of arginase-1 (Arg1), NO, and ROS, which induce apoptosis of anti-tumor CD4^+^ and CD8^+^ T cells. MDSCs also upregulate PD-L1 expression, thereby inhibiting T cell responses, and secrete immunosuppressive cytokines such as IL-10 and TGF-β, facilitating the induction of Tregs. Additionally, they promote angiogenesis, thereby supporting tumor growth (52).

Recent evidence indicates that lncRNAs play critical roles in regulating the differentiation, expansion, and immunosuppressive activity of MDSCs within the TME, thereby contributing to immune evasion in lung cancer. One such lncRNA is RUNXOR, which is markedly upregulated in both the peripheral blood and tumor-infiltrating MDSCs of lung cancer patients (53). RUNXOR negatively regulates RUNX1, a transcription factor essential for myeloid cell differentiation (54). Consistent with this, *RUNX1* expression is significantly lower in lung cancer patients than in healthy individuals, suggesting that RUNXOR-mediated repression of *RUNX1* facilitates MDSC accumulation. Functionally, silencing RUNXOR decreases the expression of Arg1, a major effector molecule of MDSC-mediated immune suppression. Collectively, these findings suggest that RUNXOR enhances the suppressive capacity of MDSCs and contributes to tumor immune evasion, highlighting its potential as a therapeutic target to restore anti-tumor immunity (53).

In contrast, **HOTAIRM1** (HOXA transcript antisense RNA myeloid-specific 1) acts as a negative regulator of MDSC-mediated immunosuppression in lung cancer (55). Its expression is markedly reduced in tumor-infiltrating MDSCs and in peripheral blood mononuclear cells (PBMCs) of lung cancer patients compared with healthy controls. Mechanistically, HOTAIRM1 upregulates HOXA1, a transcription factor promoting granulocyte differentiation, thereby limiting MDSC expansion and enhancing anti-tumor immune response (55).

Another notable lncRNA implicated in modulating MDSCs within the lung cancer TME is **MALAT1** (Metastasis-Associated Lung Adenocarcinoma Transcript 1). It is significantly downregulated in PBMCs of lung cancer patients, correlating with elevated MDSC frequencies and reduced CD8^+^ T cell activity. Knockdown of MALAT1 promotes MDSC expansion *in vitro*, suggesting that it functions as a negative regulator of tumor-promoting myeloid cells (56). These findings further underscore the contribution of lncRNAs to MDSC-driven immune suppression and their potential as therapeutic targets in lung cancer.

AK036396 (F730016J06Rik) is a murine lncRNA identified as a key regulator of PMN-MDSC function in lung tumor-bearing mice. It is highly expressed in PMN-MDSCs, where it maintains their immature state and enhances their suppressive activity. Mechanistically, AK036396 stabilizes Ficolin B (Fcnb), a protein required for PMN-MDSC development, by protecting it from ubiquitin–proteasome–mediated degradation. AK036396 knockdown therefore promotes Fcnb degradation, accelerates PMN-MDSC maturation, and reduces their immunosuppressive activity. Although a direct human ortholog of AK036396 has not been characterized, the human ortholog of Fcnb, M-ficolin, is upregulated in lung cancer and positively correlates with ARG1 expression, suggesting that the Fcnb–ARG1 axis may be functionally conserved in human disease (57).

### 2.3 T cells

CD8^+^ cytotoxic T lymphocytes (CTLs) and CD4^+^ T helper 1 (Th1) cells are central mediators of anti-tumor immunity. CTLs directly eliminate tumor cells by recognizing tumor-specific or tumor-associated antigens presented via major histocompatibility complex (MHC) class I molecules (58, 59). Upon antigen recognition, activated CTLs induce apoptosis via perforin–granzyme release or FAS–FASL interactions (58, 59). In addition to direct cytotoxicity, CTLs secrete pro-inflammatory cytokines, particularly IFN-γ, which exerts potent anti-tumor effects (60). Clinically, a high density of tumor-infiltrating CD3^+^/CD8^+^ T cells correlates with improved overall survival in various malignancies, including NSCLC, underscoring their prognostic significance (61). Th1 cells further reinforce anti-tumor immunity through IFN-γ secretion, which supports CTL activation and promotes M1 macrophage polarization, thereby fostering a pro-inflammatory and tumor-suppressive microenvironment (62). However, both CTLs and Th1 cells encounter substantial barriers within the TME. Tregs, M2 macrophages, and MDSCs—along with cytokines such as TGF-β and IL-10— inhibit their activation and effector functions (62). In addition, immune checkpoint molecules (e.g., PD-L1, CTLA-4) induce T cell exhaustion, while tumor cells evade recognition through antigen loss or MHC class I downregulation (63, 64). Metabolic constraints, such as hypoxia and nutrient deprivation, further compromise CTL and Th1 cell functions within the TME (65).

Given their pivotal roles in anti-tumor defense, increasing attention has been directed toward understanding how lncRNAs modulate T cell differentiation, activation, and exhaustion in lung cancer. Several lncRNAs have emerged as key regulators of T cell functionality, shaping the composition and cytotoxic potential of TILs. Among the lncRNAs identified, **NKILA** and **lncNDEPD1** have emerged as key regulators of CD8^+^ T cell and Th1 cell function in lung cancer, exerting distinct effects on T cell survival and immune checkpoint regulation.

**NKILA** (NF-κB Interacting LncRNA) plays a critical role in regulating the survival of tumor-infiltrating CTLs and Th1 cells. It is strongly induced in activated T cells via the IFN-γ–JAK–STAT1 pathway following T cell receptor (TCR) stimulation. Mechanistically, TCR signaling increases intracellular calcium, triggering calmodulin-mediated displacement of histone deacetylases (HDACs), thereby allowing STAT1 to activate NKILA transcription. Once expressed, NKILA binds to and inhibits NF-κB, a key transcription factor that normally promotes T cell survival. This inhibition sensitizes CTLs and Th1 cells to activation-induced cell death (AICD), thereby depleting them within the TME. In contrast, immunosuppressive subsets such as Tregs and Th2 cells, which express low levels of NKILA, remain resistant to AICD, promoting an immunosuppressive milieu. In NSCLC patients, NKILA is highly expressed in tumor-specific CTLs, and elevated NKILA levels correlate with reduced CTL infiltration and shorter disease-free survival, indicating its negative prognostic impact. Functional studies further demonstrate that silencing NKILA enhances CTLs survival, tumor infiltration, and anti-tumor responses *in vivo*, whereas forced expression of NKILA in Tregs or Th2 cells renders these cells susceptible to AICD (66). Collectively, NKILA functions as an intrinsic immune checkpoint that limits anti-tumor immunity by promoting apoptosis of tumor-reactive T cells in lung cancer.

**lncNDEPD1** modulates immune checkpoint expression in CD8+ T cells. In peripheral blood of NSCLC patients, lncNDEPD1 expression positively correlates with *PDCD1* (PD-1) mRNA levels, highlighting its role in T cell exhaustion. Localized in the cytoplasm, lncNDEPD1 acts as a ceRNA and sponges miR-3619-5p, thereby preventing miRNA-mediated degradation of PDCD1 mRNA and sustaining elevated PD-1 expression in CD8^+^ T cells. Experimental knockdown of lncNDEPD1 in chimeric antigen receptor T (CAR-T) cells significantly reduces PD-1 expression and enhances their cytotoxicity against PD-L1–positive tumors *in vivo* (67). These findings identify lncNDEPD1 as a positive regulator of PD-1–mediated T cell exhaustion and suggest that its inhibition could potentiate the efficacy of T cell–based immunotherapies in lung cancer.

## 3 Tumor cell-derived lncRNAs regulating the lung tumor immune microenvironment

**Figure 4:**
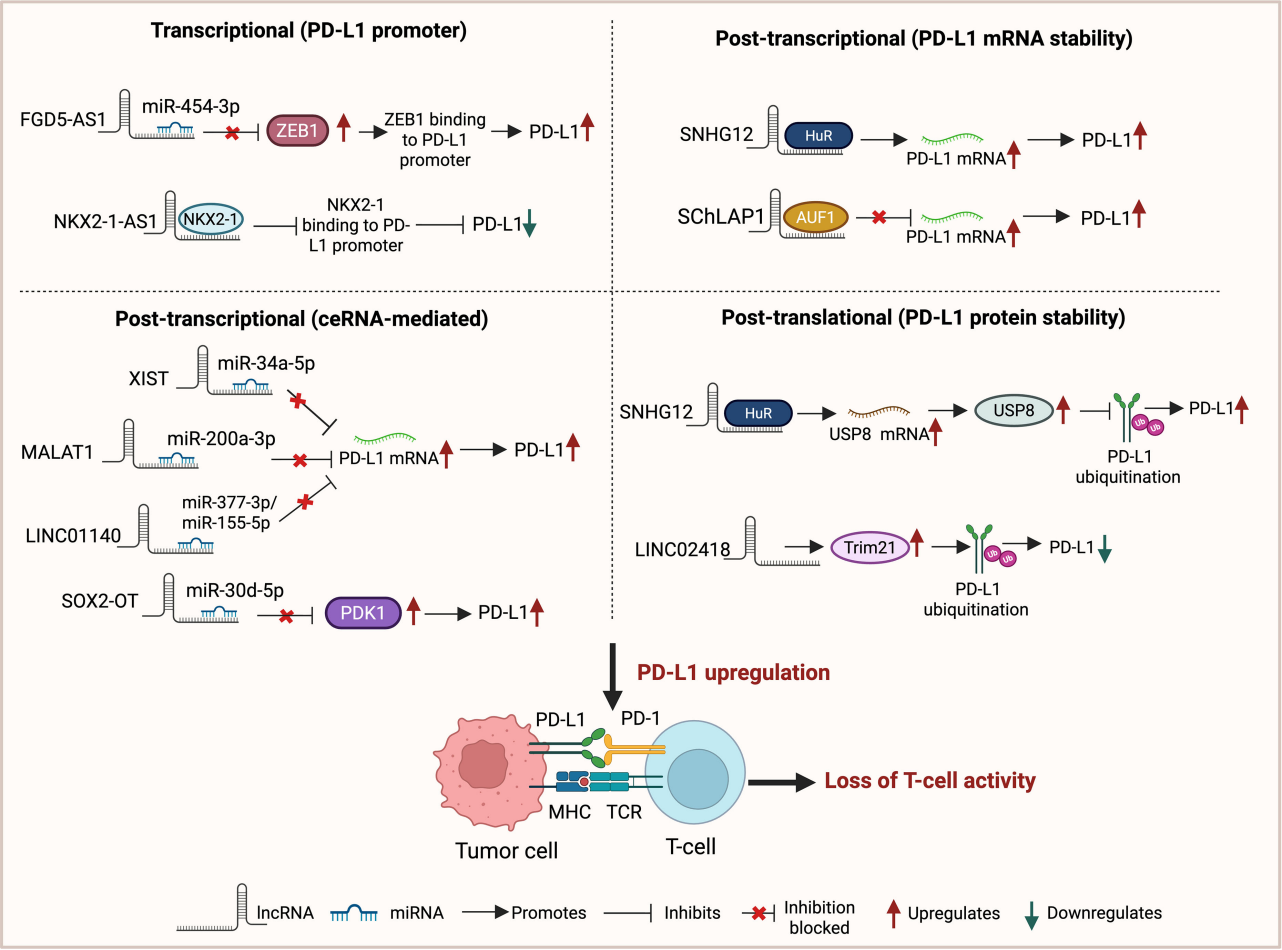
Tumor-derived lncRNAs regulating PD-L1/PD-1 immune checkpoint axis. Tumor-derived lncRNAs modulate PD-L1 expression at four distinct regulatory levels: transcriptional, post-transcriptional via RNA-binding protein interaction, post-transcriptional via ceRNA-mediated miRNA sequestration, and post-translational. Transcriptional: FGD5-AS1 sponges miR-454-3p to derepress ZEB1, which binds the PD-L1 promoter and drives PD-L1 transcription. NKX2-1-AS1 binds NKX2-1, preventing it from binding the PD-L1 promoter and thereby inhibiting PD-L1 transcription. Post-transcriptional (via RNA-binding protein): SNHG12 interacts with HuR to stabilize PD-L1 mRNA, while SChLAP1 binds AUF1 to block AUF1-mediated PD-L1 mRNA degradation. Post-transcriptional (ceRNA-mediated): XIST, MALAT1, and LINC01140 sponge miR-34a-5p, miR-200a-3p, and miR-377-3p/miR-155-5p, respectively, all derepressing PD-L1 mRNA. SOX2-OT sponges miR-30d-5p to derepress PDK1, promoting PD-L1 expression. Post-translational (PD-L1 protein stability): SNHG12 also stabilizes USP8 mRNA via HuR; elevated USP8 deubiquitinates PD-L1 to prevent its degradation. Conversely, LINC02418 enhances Trim21-mediated PD-L1 ubiquitination and degradation. Collectively, dysregulation of these lncRNAs alters PD-L1 surface expression, modulating PD-L1/PD-1 engagement with cytotoxic T cells. Created with BioRender.com.

**Figure 5:**
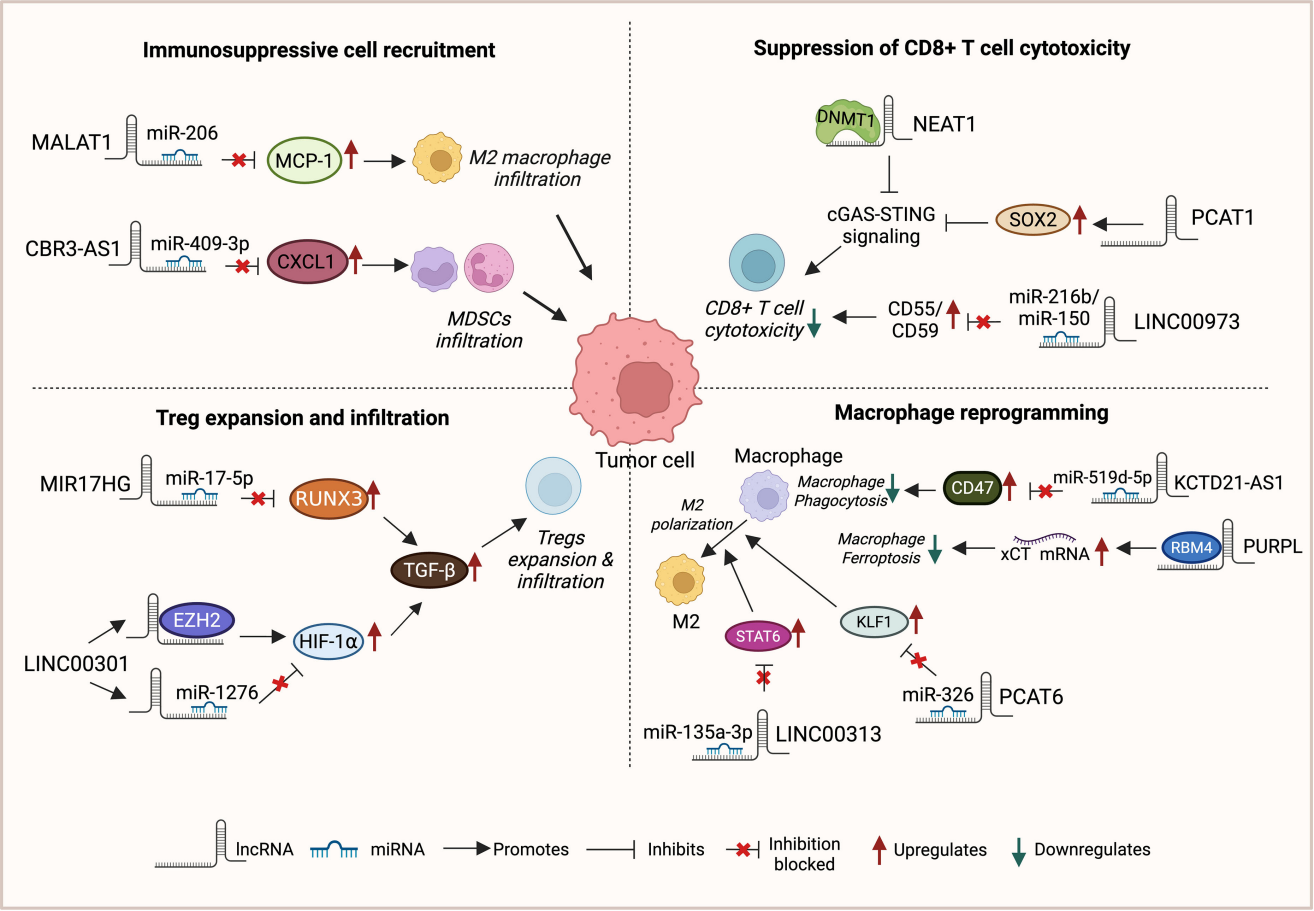
Tumor-derived lncRNAs regulating the lung tumor immune microenvironment. Tumor-derived lncRNAs establish an immunosuppressive tumor microenvironment through four functional outcomes. Immunosuppressive cell recruitment: MALAT1 sponges miR-206 to derepress MCP-1, recruiting M2 macrophages, while CBR3-AS1 sponges miR-409-3p to derepress CXCL1, recruiting myeloid-derived suppressor cells (MDSCs). Suppression of CD8^+^ T cell cytotoxicity: NEAT1 (via DNMT1) and PCAT1 (via SOX2) independently silence cGAS-STING signaling, while LINC00973 sponges miR-216b/miR-150 to derepress the complement regulators CD55/CD59 — collectively impairing CD8^+^ T cell-mediated antitumor responses. Treg expansion and infiltration: MIR17HG sponges miR-17-5p to derepress RUNX3, and LINC00301 elevates HIF-1α through dual mechanisms — EZH2-mediated stabilization and miR-1276 sponging — both upregulating TGF-β to drive Treg expansion and infiltration. Macrophage reprogramming: KCTD21-AS1 sponges miR-519d-5p to derepress CD47, suppressing macrophage phagocytosis. PURPL binds the RNA-binding protein RBM4 to stabilize xCT mRNA, blocking macrophage ferroptosis. PCAT6 (via miR-326/KLF1) and LINC00313 (via miR-135a-3p/STAT6) promote M2 macrophage polarization. Collectively, these lncRNAs remodel the immune microenvironment toward an immunosuppressive state, supporting tumor growth, proliferation, and invasiveness. Created with BioRender.com.

Tumor cells actively shape their immune microenvironment through diverse molecular mechanisms that facilitate immune evasion, tumor progression, and therapy resistance. Among these regulators, lncRNAs have emerged as critical players that modulate immune responses either by altering immune cell infiltration and activation or by reprogramming tumor-intrinsic signaling pathways. Tumor cell-derived lncRNAs regulate immune checkpoint expression, cytokines, and chemokines secretion, and antigen presentation, promoting an immunosuppressive niche conducive to tumor growth. In this section, we discuss key tumor-derived lncRNAs identified in NSCLC, focusing on their expression status, molecular mechanisms, biological effects, prognostic implications, and therapeutic relevance (**Table 2**; **Figures 4**, **5**).

**Table 2:** Summary of tumor cell-derived lncRNAs regulating the lung tumor immune microenvironment in NSCLC.

LncRNA	Sample type	Status in cancer	Prognosis	Effects	Mechanism	Functional validation	Level of evidence	Reference
SNHG12	Tumor tissue	Upregulated	Poor prognosis	Upregulates PD-L1 expression promoting immune evasion	SNHG12 binds HuR, stabilizing PD-L1 and USP8 mRNA, increasing PD-L1 expression	*In vitro*- siRNA knockdown, *in vivo*- mouse xenograft tumor model	Clinical + Preclinical	(68)
SChLAP1	Tumor tissue and serum	Upregulated	Poor prognosis	Promotes proliferation, migration, invasion of tumor cells; inhibits CD8^+^ T cell function	Binds AUF1, increasing PD-L1 mRNA stability and upregulating PD-L1 expression	*In vitro*- knockdown and overexpression assay, *in vivo*- tumor/metastasis xenograft model	Clinical + Preclinical	(69)
XIST	Tumor tissue and serum	Upregulated	Not reported	Promotes viability, apoptosis, migration, and invasion of lung cancer cells; suppress CD8+ T cells; promotes M2 polarization	Sponges miR-34a-5p to upregulate PD-L1	*In vitro*- knockdown and overexpression assay	Clinical + Preclinical	(70–72)
FGD5-AS1	Tumor tissue	Upregulated	Poor prognosis	Promotes angiogenesis and immune evasion; enhances proliferation and invasion of tumor cells	Sponges miR-454-3p → upregulates ZEB1 → activates VEGFA and PD-L1 expression	*In vitro* -knockdown and overexpression assay, *In vivo*-xenograft tumor mice model	Clinical + Preclinical	(73)
NKX2-1-AS1	Tumor tissue	Upregulated	Not reported	Tumor suppressive role- Inhibits PD-L1 expression, Reduces Tumor cell migration, no effect on proliferation or apoptosis	Interacts with NKX2–1 protein → prevents its binding to PD-L1 promoter → downregulates PD-L1	*In vitro*- siRNA-mediated knockdown	Clinical + Preclinical + In silico	(74)
LINC02418	Tumor tissue	Not reported	Favorable prognosis	Downregulates PD-L1 expression, restores CD8+ T cell-mediated anti-tumor immunity	Enhances Trim21 mediated ubiquitination and degradation of PD-L1	*In vitro*- knockdown and overexpression assay, *in vivo*- syngeneic mouse model	Clinical + Preclinical + In silico	(75)
LINC01140	Tumor tissue	Upregulated	Poor prognosis	Promotes PD-L1 expression, enhances immune evasion; promotes tumor cell proliferation, migration, invasion, cisplatin resistance	Sponges miR-33a-5p/miR-33b-5p (derepresses c-Myc) and miR-377-3p/miR-155-5p (derepresses PD-L1)	*In vitro* — knockdown assays; *in vivo* —xenograft in SCID mice	Clinical + Preclinical	(76)
SOX2-OT	Tumor tissue (TCGA dataset analysis)	Upregulated	Not reported	Promotes tumor proliferation, immune evasion, and CD8^+^ T cell apoptosis	Sponges miR-30d-5p → upregulates PDK1 → activates mTOR signaling → induces PD-L1 expression	I*in vitro*- shRNA mediated knockdown assay, *in vivo*-mouse xenograft tumor model	Preclinical+ In silico	(77)
MALAT1	Tumor tissue and plasma	Upregulated	Poor prognosis	Promotes M2 macrophage recruitment; upregulates PD-L1 expression, promotes immune evasion; enhances tumor growth and invasiveness	(1) Sponges miR-206 → upregulates MCP-1 → recruits M2 macrophages (2) Sponges miR-200a-3p → upregulates PD-L1	*In vitro*- overexpression and knockdown, *in vivo*- xenograft tumor mice models	Clinical + Preclinical + In silico	(78–80)
NEAT1	Tumor tissue	Upregulated	Associated with advanced stage of disease	Promotes lung cancer cells viability and migratory activity and inhibiting CD8+ T cell infiltration	NEAT1 binds DNMT1, silencing p53 and cGAS-STING pathway	*In vitro*- shRNA knockdown, and *in vivo* – mouse syngeneic tumor model	Clinical + Preclinical + In silico	(81)
LINC00973	Tumor tissue	Upregulated	Poor prognosis	Reduces complement activation, inhibits CD8+ T cell function,immune escape	EGFR/Wnt/β-catenin → LINC00973 → sponging miR-216b/miR-150 → ↑CD55/CD59	*In vitro*- knockdown and *in vivo*-mouse xenograft tumor model	Clinical + Preclinical + In silico	(82, 83)
PCAT1	Tumor tissue	Upregulated	Poor prognosis	Promotes proliferation, metastasis, immune evasion, and radioresistance	Activates SOX2 → suppresses cGAS/STING pathway → inhibits type-1 interferon (IFN-I) response and T cell activation	*In vitro*-siRNA-mediated knockdown assays and *in vivo*- mouse xenograft tumor model	Clinical + Preclinical + In silico	(84)
LINC00892	Tumor tissue	Downregulated	Poor prognosis (Low LINC00892 expression associated with poor OS, low DSS, low PFI)	Inhibits proliferation, migration, EMT of tumor cells; promotes apoptosis; enhances CD4^+^ and CD8^+^ T cell infiltration	Suppresses EMT by upregulating E-cadherin and downregulating N-cadherin, vimentin, and Slug	*In vitro* - overexpression assay and *in vivo* -mouse xenograft tumor model	Clinical + Preclinical + In silico	(85)
CBR3-AS1	Tumor tissue	Upregulated	Poor prognosis (High CBR3-AS1 associated with poor OS and poor DFS)	Promotes proliferation, invasion, radioresistance of NSCLC cells; recruits MDSCs; suppresses CD4+ T and CD8+ T cells infiltration	Sponges miR-409-3p to upregulate CXCL1	*In vitro*- overexpression and knockdown assays, *In vivo*-mouse xenograft tumor model	Clinical + Preclinical + In silico	(86, 87)
LINC00301	Tumor tissue	Upregulated	Poor prognosis (high LINC00301 expression associated with low OS)	Promotes proliferation, migration, invasion; suppresses apoptosis of NSCLC tumor cells; increases Tregs, decreases CD8^+^ T cell infiltration	LINC00301 activates HIF-1α by silencing EAF2 (via EZH2) and sponging miR-1276; HIF-1α induces TGF-β	*In vitro*- knockdown and overexpression assays; *in vivo*- mouse xenograft tumor model	Clinical + Preclinical + In silico	(88)
MIR17HG	Tumor tissue	Upregulated	Not reported	Promotes tumor growth and Treg-mediated immune escape	Sponges miR-17-5p to upregulate RUNX3	*In vitro*- knockdown assays and *In vivo*- mouse tumor xenograft model	Clinical + Preclinical + In silico	(89)
NPSR1-AS1	Tumor tissue (TCGA dataset analysis)	Upregulated	Poor prognosis (high NPSR1-AS1 expression associated with low OS and DSS)	Negatively correlated with cytotoxic CD8+ T cells, DCs, macrophages and Th17 cells	Not reported	Not reported	In silico	(90)
PVT1	Tumor Tissue, PMN-MDSCs (murine)	Upregulated	Poor prognosis (High PVT1 associated with low OS)	Enhances tumor cell proliferation, migration and invasion; promotes PMN-MDSCs immunosuppressive function	Not reported	*In vitro*- knockdown assay and *in vivo*- mouse tumor model	Clinical+ Preclinical	(91, 94)
Snhg6	Tumor tissue, M-MDSCs (murine)	Upregulated	Poor prognosis (High Snhg6 associated with low OS)	Promotes tumor cell growth,migration and invasion; promotes M-MDSCs differentiation	Tumor cell-Sponges miR-485-3p to upregulate VPS45 via SPI1/Snhg6 axis; M-MDSCs- promotes EZH2 ubiquitination and proteasomal degradation	*In vitro*- siRNA knockdown; *in vivo*- mouse xenograft tumor model	Clinical+ Preclinical	(95, 96)
linc-EPHA6-1	A549 lung cancer cell derived exosomes	Not reported	Not reported	Promotes NK cell- mediated cytotoxicity	Sponges hsa-miR-4485-5p upregulating NKp46	*In vitro*- overexpression assay	Preclinical	(97)
KCTD21-AS1	Tumor tissue	Upregulated	Poor prognosis(high expression associated with low OS)	Promotes tumor cell proliferation, inhibits macrophage mediated phagocytosis	Sponges miR-519d-5p → upregulates CD47 (immune evasion) and TIPRL (autophagy suppression)	*In vitro*- knockdown and overexpression assay;*in vivo*- mouse xenograft tumor model	Clinical + Preclinical + In silico	(98)
LINC00313	Tumor tissue and serum	Upregulated	Poor prognosis (high LINC00313 associated with low OS)	Promotes tumor progression, induces M2 macrophage polarization	Sponges miR-4429 in tumor cells; Sponges miR-135a-3p in TAMs; upregulates STAT6	*In vitro*- knockdown and overexpression assay;*in vivo*- mouse xenograft tumor model	Clinical + Preclinical + In silico	(99, 100)
PCAT6	Tumor tissue	Upregulated	Not reported	Promotes proliferation, migration, invasion, EMT of tumor cells; induces M2 macrophage polarization	Sponges miR-330-5p to enhance tumor invasiveness; sponges miR-326 in macrophages to upregulate KLF1	*In vitro* - knockdown assays and *in vivo*- mouse xenograft tumor model	Clinical + Preclinical + In silico	(101, 102)
HOXC-AS2	Tumor Tissue	Upregulated	Not reported	Promotes tumor cell proliferation, migration, and EMT; induces M2 macrophage polarization	Interacts with HOXC13 to promote EMT; sponged into exosomes and transferred to macrophages where it binds STAT1, suppressing SOCS1/CIITA signaling to drive M2 polarization	*In vitro*- knockdown/overexpression assays; *in vivo* mouse xenograft tumor model	Clinical+ Preclinical	(103, 104)
LINC01116	Tumor Tissue (TCGA dataset analysis); patient-derived circulating EVs	Upregulated	Poor prognosis	Promotes tumor cell proliferation, migration, invasion; induces macrophage M2 polarization	ceRNA — sponges miR-3614-5p to upregulate ARHGAP1	*In vitro*- knockdown assay; *in vivo*- mouse tumor model	Clinical + Preclinical + In silico	(105)
SNHG16	Tumor Tissue and circulating EVs	Upregulated	Poor prognosis	Promotes tumor cell proliferation, EMT, cancer stem cell properties; induces macrophage M2 polarization	ceRNA — sponges miR-132-3p to upregulate KIF5A	*In vitro*- knockdown assay; *in vivo*- mouse tumor model	Clinical + Preclinical+ In silico	(106)
PURPL	Tumor tissue (TCGA dataset analysis)	Upregulated	Not reported	Promotes M2 macrophage polarization	Binds RBM4 to stabilize xCT mRNA → increases xCT expression → inhibits ferroptosis in macrophages	*In vitro*- overexpression assay	Preclinical+ In silico	(107)

The table summarizes tumor-derived lncRNAs characterized in NSCLC, including the sample type assessed, expression status in cancer, associated prognosis, functional effects, molecular mechanisms, validation approaches, level of supporting evidence, and references.

Level of Evidence: *Clinical* — evidence from human patient samples; *Preclinical* — functional characterization in cell lines or animal models; *In silico* — computational analysis of public datasets (e.g., TCGA, GEO) without accompanying wet-lab validation. Studies combining multiple approaches are labelled accordingly (e.g., *Clinical + Preclinical*, *Clinical + Preclinical + In silico*).

OS, overall survival; DFS, disease-free survival; DSS, disease-specific survival; PFI, progression-free interval; EVs, extracellular vesicles; MDSCs, myeloid-derived suppressor cells; PMN-MDSCs, polymorphonuclear myeloid-derived suppressor cells; M-MDSCs, monocytic myeloid-derived suppressor cells; TAMs, tumor-associated macrophages; Tregs, regulatory T cells; EMT, epithelial-mesenchymal transition; ceRNA, competing endogenous RNA; DCs, dendritic cells; NK cells, natural killer cells; PD-L1, programmed death-ligand 1; TCGA, The Cancer Genome Atlas.

### 3.1 Tumor-derived lncRNAs regulating PD-L1/PD-1 immune checkpoint axis

**SNHG12** (Small Nucleolar RNA Host Gene 12) is significantly overexpressed in NSCLC tumor tissues and positively correlates with PD-L1 expression. High SNHG12 expression correlates with shorter OS in NSCLC patients. Mechanistically, SNHG12 interacts with the RNA-binding protein HuR, enhancing the stability of PD-L1 and USP8 mRNAs. Elevated USP8 expression prevents PD-L1 degradation through deubiquitination, resulting in sustained PD-L1 expression that inhibits CD8^+^ T cell-mediated anti-tumor activity. Silencing SNHG12 reduces PD-L1 levels, restores T cell activity, and inhibits tumor growth, highlighting its potential as a therapeutic target to enhance immunotherapy in NSCLC (68).

SChLAP1 (Second Chromosome Locus Associated with Prostate-1) is elevated in NSCLC tissues and patients’ serum. High SChLAP1 expression correlates with poor OS. It promotes tumor progression and immune evasion through the PD-1/PD-L1 axis. By directly binding RNA-binding protein AUF1, SChLAP1 prevents AUF1-mediated PD-L1 mRNA degradation, increasing PD-L1 stability and expression on tumor cells (69).

XIST is significantly upregulated in both tumor tissues and serum of NSCLC patients compared with healthy controls (70). Functionally, XIST acts as a ceRNA that sponges miR-34a-5p, which is downregulated in NSCLC and normally represses PD-L1 expression. By sequestering miR-34a-5p, XIST upregulates PD-L1, thereby promoting tumor cell proliferation, invasion, and resistance to CD8^+^ T cell–mediated cytotoxicity (71). Beyond its tumor-intrinsic role, *XIST* also contributes to immune modulation in the TME (72). Preclinical studies demonstrate that *XIST* expression in macrophages is transcriptionally induced by TCF-4, which binds directly to its promoter and thereby promotes M2 macrophage polarization. Additionally, tumor-conditioned media upregulate *XIST* in macrophages, suggesting that tumor-derived factors may drive immunosuppression through the TCF-4/*XIST* axis (72).

**FGD5-AS1**, upregulated in NSCLC tissues and correlated with poor OS, sponges miR-454-3p, derepressing its downstream target, ZEB1 (Zinc finger E-box-binding homeobox 1). ZEB1 enhances the expression of VEGFA (vascular endothelial growth factor A), a key driver of tumor angiogenesis, and PD-L1. Experimental knockdown of FGD5-AS1 in NSCLC cell lines results in reduced proliferation, invasion, angiogenic potential, and improved CD8^+^ T cell activity. *In vivo* studies further confirm that silencing FGD5-AS1 suppresses tumor growth, diminishes VEGFA and PD-L1 expression, and restores anti-tumor immune responses (73).

**NKX2-1-AS1** is an antisense lncRNA that partially overlaps the NKX2-1/TTF1 locus at chromosome 14q13.3 – a region frequently amplified in lung adenocarcinomas (74). Although both NKX2–1 and NKX2-1-AS1 are upregulated in lung adenocarcinoma relative to normal lung tissue, their expression is independently regulated. Functionally, NKX2-1-AS1 acts as a tumor-suppressive lncRNA by attenuating tumor immune evasion and migratory potential. It acts in trans to repress genes involved in cell adhesion and PD-L1/PD-1 signaling pathways. Mechanistically, NKX2-1-AS1 inhibits PD-L1 expression by interfering with NKX2–1 protein binding to the PD-L1 promoter. Additionally, it suppresses tumor cell migration and wound-healing ability without affecting proliferation or apoptosis (74).

**LINC02418** expression negatively correlates with PD-L1 levels and positively correlates with CD8+ T cell infiltration in NSCLC patients, predicting favorable clinical outcomes (75). Mechanistically, LINC02418 enhances Trim-21 mediated ubiquitination and proteasomal degradation of PD-L1, thereby reducing PD-L1 expression on tumor cells and enhancing T cell mediated anti-tumor immunity. Both *in vitro* and *in vivo* syngeneic models confirm that LINC02418 enhances the efficacy of anti-PD-L1 therapy by promoting T cell-mediated tumor cell apoptosis, highlighting its potential as both a predictive biomarker of ICI response and a therapeutic target in NSCLC (75).

**LINC01140** is highly expressed in NSCLC tumor tissues and cell lines, and its high expression is associated with poor OS (76). It promotes tumor progression through a dual ceRNA mechanism, sponging miR-33a-5p and miR-33b-5p to derepress c-Myc, thereby enhancing proliferation, migration, invasion, and resistance to cisplatin-induced apoptosis. In parallel, LINC01140 sponges miR-377-3p and miR-155-5p, both of which share PD-L1 as a downstream target; their sequestration results in elevated PD-L1 expression and consequent immune evasion. *In vivo*, LINC01140 knockdown combined with cytokine-induced killer cell administration suppressed subcutaneous lung cancer xenograft growth and reduced PD-L1 levels in SCID mice, suggesting that LINC01140 may represent a therapeutically actionable target in lung cancer (76).

**SOX2-OT** (SOX2 overlapping transcript) is overexpressed in NSCLC tumor cells as identified by bioinformatic analysis of TCGA datasets, followed by validation in NSCLC cell lines (77). SOX2-OT contributes to immune evasion and tumor progression through the SOX2-OT/miR-30d-5p/PDK1 regulatory axis. Specifically, SOX2-OT sponges miR-30d-5p, which leads to elevated levels of its downstream target, PDK1—a kinase activating the pro-tumorigenic mTOR signaling pathway. Activation of this pathway promotes the expression of PD-L1 on tumor cells, subsequently impairing CD8^+^ T cell-mediated antitumor immunity (77). However, due to the lack of direct clinical tissue validation, further studies are needed to confirm the translational relevance of SOX2-OT in lung cancer patient samples.

### 3.2 Tumor-derived lncRNAs promoting an immunosuppressive microenvironment

This section summarizes tumor-derived lncRNAs that suppress CD8+ T cell infiltration and cytotoxic function while promoting the recruitment of immunosuppressive cell populations, including Tregs, MDSCs, and M2 macrophages.

**MALAT1** promotes NSCLC progression via multiple immune-modulatory mechanisms (78, 79). High MALAT1 expression in early-stage NSCLC patients is associated with poor prognosis (80). MALAT1 is elevated in both plasma and tumor tissues of lung cancer patients compared to healthy controls and is further elevated in tumors of African American patients relative to White American patients, showing its potential role in contributing to racial disparities in lung cancer outcomes (79). Mechanistically, MALAT1 acts as a ceRNA by sponging miR-206, thereby upregulating MCP-1 (CCL2), a chemokine involved in the recruitment of immunosuppressive macrophages. High expression of MALAT1 correlates with increased expression of MCP-1 and M2 macrophage markers (CD68, CD163, CD206) (79). MALAT1 also modulates the miR-200a-3p/PD-L1 axis to enhance PD-L1-mediated immune escape (78). MALAT1 overexpression promotes tumor cell proliferation and migration, and resistance to anti-tumor immunity (78, 79).

**NEAT1** (Nuclear Enriched Abundant Transcript 1) is prominently overexpressed in NSCLC tumor tissues and correlates with advanced TNM stage and lymph node metastasis (81). NEAT1 binds to DNMT1, leading to hypermethylation and transcriptional silencing of p53 and the cGAS-STING pathway. By inhibiting p53, NEAT1 promotes the viability and migratory abilities of lung cancer cells. By inhibiting cGAS-STING, NEAT1 suppresses the production of interferons and pro-inflammatory cytokines, thereby reducing CD8^+^ cytotoxic T cell infiltration into the TME. Silencing NEAT1 impairs tumor cell proliferation and invasion, and enhances T cell–mediated antitumor responses *in vivo* (81). These findings highlight NEAT1 as a key modulator of the epigenetic landscape that controls innate immune sensing and T cell recruitment in lung cancer.

**LINC00973** is upregulated in NSCLC tumor tissues and correlates with poor prognosis, as supported by both TCGA datasets and clinical samples (82). Transcriptomic network analysis revealed a LINC00973–miRNA–mRNA ceRNA axis involving 15 miRNAs and over 200 target mRNAs enriched in oncogenic pathways such as PI3K–Akt, MAPK, TGF-β, and cell cycle signaling, suggesting roles in tumor proliferation, migration, and endothelial activation (82). Upstream regulation of LINC00973 involves EGFR/Wnt/β-catenin signaling, which enhances its expression (83). Mechanistically, LINC00973 sponges miR-216b and miR-150, releasing suppression of CD55 and CD59, two complement regulatory proteins that inhibit complement-mediated cytotoxicity. Elevated *CD55*/*CD59* expression impairs CD8^+^ T cell–mediated immune responses and correlates inversely with M1 macrophage and CD8^+^ T cell infiltration, predicting poorer clinical outcomes (83).

PCAT1 (Prostate Cancer Associated Transcript 1) is significantly upregulated in NSCLC and promotes tumor progression and immune evasion. High PCAT1 expression is associated with poor OS in NSCLC patients (84). Mechanistically, PCAT1 enhances SOX2 transcription, which in turn suppresses cGAS expression and downstream STING-mediated type I interferon response— a pathway critical for cytotoxic T cell activation. This enables tumor immune escape and reduces the efficacy of radiotherapy. Functionally, PCAT1 knockdown restrains NSCLC cell proliferation and metastasis while enhancing apoptosis and immune cell infiltration. Combined inhibition of PCAT1 and SOX2 synergistically restores antitumor immunity and sensitizes tumors to radiotherapy (84).

**LINC00892** has been identified as a tumor-suppressive and prognostic lncRNA in lung adenocarcinoma (LUAD). Its expression is markedly lower in LUAD tissues than in normal lung tissue and is associated with poorer OS, disease-specific survival (DSS), and progression-free interval (PFI), particularly in younger and early-stage patients (85). Its expression is positively associated with CD4^+^ and CD8^+^ T cell infiltration and negatively with tumor-promoting Th2 cells, suggesting a role in modulating the tumor immune microenvironment. LINC00892 overexpression suppresses LUAD cell proliferation, migration, and EMT, while promoting apoptosis. It suppresses EMT by upregulating the expression of E-cadherin and downregulating N-cadherin, vimentin, and Slug. *In vivo* xenograft models confirm that LINC00892 overexpression reduces tumor growth and metastasis, concomitant with increased CD4^+^ and CD8^+^ T cell infiltration (85).

**CBR3-AS1** is another tumor-derived lncRNA implicated in NSCLC immune evasion and radioresistance (86, 87). Its expression is significantly elevated in NSCLC tissues and correlates with poor survival. In radioresistant tumors, CBR3-AS1 accumulation is driven by methyltransferase RBM15-mediated m6A modifications and stabilized via m6A reader IGF2BP3 (87). Functionally, CBR3-AS1 acts as a ceRNA by sponging miR-409-3p, thereby upregulating CXCL1, which facilitates recruitment of MDSCs. The resulting immunosuppressive microenvironment impairs CD4+ and CD8+ T cell responses and contributes to radiotherapy resistance (87).

LINC00301 is highly expressed in NSCLC and is associated with poor prognosis and increased tumor aggressiveness (88). LINC00301 promotes proliferation, invasion, and survival through dual mechanisms in the nucleus and cytoplasm. In the nucleus, it interacts with EZH2 to promote H3K27me3-mediated repression of EAF2 and stabilizes HIF1α; in the cytoplasm, it acts as a ceRNA sponging miR-1276, further increasing HIF1α levels. Elevated HIF1α induces TGF-β, promoting Tregs expansion and inhibiting CD8^+^ T cell infiltration (88).

MIR17HG (miR-17–92 cluster host gene) is markedly upregulated in NSCLC and drives tumor growth and immune evasion by enhancing Treg-mediated suppression (89). MIR17HG sponges miR-17-5p to upregulate RUNX3, leading to increased IL-10, TGF-β, IL-4, and VEGF-A secretion while reducing IFN-γ production. Silencing MIR17HG suppresses tumor cell proliferation, reduces Treg recruitment, and restores anti-tumor immunity (89).

**NPSR1-AS1** is significantly upregulated in LUAD tissues compared to normal lung tissues, as identified through bioinformatic analysis of the TCGA datasets (90). Patients with high NPSR1-AS1 expression exhibit significantly lower OS and DSS compared with those showing low expression. Functional enrichment analysis indicates that NPSR1-AS1 participates in multiple tumor-promoting signaling pathways. Moreover, immune infiltration analysis reveals a strong negative correlation between NPSR1-AS1 expression and the abundance of cytotoxic and antigen-presenting immune cells, including CD8^+^ T cells, dendritic cells, macrophages, and Th17 cells (90). This suggests a potential role of NPSR1-AS1 in shaping an immunosuppressive tumor microenvironment. However, its role requires further experimental validation through *in vitro* and *in vivo* studies.

PVT1 (Plasmacytoma Variant Translocation 1) is a well-characterized oncogenic lncRNA, whose expression is upregulated in NSCLC tissues compared with normal adjacent tissues and positively correlates with histological grade and lymph node metastasis (91). High PVT1 expression is associated with lower OS in NSCLC patients (91). It promotes the proliferation, migration and invasion of tumor cells (91). Located on chromosome 8q24 in humans—often co-amplified with the oncogene *MYC*—PVT1 contributes to tumorigenesis through synergistic activation with *MYC* (92, 93). Beyond its tumor cell-intrinsic oncogenic roles, PVT1 also modulates the immune microenvironment through regulation of MDSCs (94). In mice, the homologous gene Pvt1 resides on chromosome 15 and retains conserved oncogenic functions despite limited sequence similarity—a common feature of many lncRNAs (94). It promotes an immunosuppressive microenvironment by enhancing the function of PMN-MDSCs (referred to as G-MDSCs in the original study). *Pvt1* expression is elevated in tumor-derived PMN-MDSCs compared with splenic counterparts, and its knockdown diminishes PMN-MDSC suppressive activity, increases CD8^+^ T and Th1 cell infiltration, and impedes tumor growth *in vivo* (94). Furthermore, *Pvt1* is upregulated under hypoxia via HIF-1α, linking its activity to metabolic adaptation. Collectively, these findings position *PVT1/Pvt1* as a potential target for disrupting MDSC-mediated immunosuppression in lung cancer (94).

Snhg6 (Small Nucleolar RNA Host Gene 6) is significantly upregulated in NSCLC tumor tissues and cell lines compared to normal controls, and high expression negatively correlates with OS in NSCLC patients (95). Mechanistically, Snhg6 exerts oncogenic effects through the SPI1/SNHG6/miR-485-3p/VPS45 axis, where transcription factor SPI1 drives Snhg6 expression, which in turn sponges miR-485-3p to upregulate VPS45, promoting NSCLC cell growth, migration, and invasion (95). Beyond its tumor cell-intrinsic role, Snhg6 has been implicated in the regulation of M-MDSC differentiation within the lung TME (96). In Lewis lung carcinoma xenografts, *Snhg6* is enriched in tumor-derived MDSCs and facilitates M-MDSC development by promoting EZH2 ubiquitination and proteasomal degradation, thereby relieving EZH2-mediated repression of M-MDSC differentiation (96). Targeting Snhg6 may thus offer a means to limit M-MDSC expansion and potentially attenuate MDSC-driven immunosuppression in NSCLC.

linc-EPHA6–1 is enriched in exosomes secreted by IFNβ-treated A549 lung cancer cells and can be internalized by NK cells. Within NK cells, it acts as a ceRNA that sponges hsa-miR-4485-5p, leading to the upregulation of NKp46, a critical receptor for NK cell cytotoxicity. Overexpression of linc-EPHA6–1 enhances NK-mediated killing of both A549 tumor and virus-infected cells, indicating its role in promoting antitumor and antiviral immunity (97). These findings support its potential as an exosome-mediated immunomodulatory lncRNA in NSCLC.

### 3.3 Tumor-derived lncRNAs reprogramming macrophage function

KCTD21-AS1 is significantly upregulated in NSCLC tumor cells and is associated with poor patient survival (98). Functionally, KCTD21-AS1 promotes tumor progression by modulating both macrophage activity and tumor cell autophagy. Its expression is stabilized by METTL14-mediated m6A modification, which enhances its oncogenic effects. Mechanistically, KCTD21-AS1 acts as a ceRNA for miR-519d-5p, thereby relieving repression of CD47 and TIPRL. CD47 serves as a “don’t eat me” signal that inhibits macrophage-mediated phagocytosis, while TIPRL suppresses autophagy, promoting tumor cell survival. KCTD21-AS1 enhances NSCLC cell proliferation and metastasis, whereas overexpression of miR-519d-5p suppresses these effects (98).

LINC00313 is upregulated in tumor tissues and serum of NSCLC patients compared to healthy controls, while its direct target miR-4429 is downregulated (99). High LINC00313 expression is associated with poor prognosis, and both LINC00313 and miR-4429 show strong potential as diagnostic and prognostic biomarkers, as their levels are abnormally expressed in NSCLC patients (99). Beyond its biomarker value, LINC00313 modulates the TIME. LINC00313-loaded exosomes secreted by tumor cells are internalized by macrophages, inducing M2 polarization. LINC00313 acts as a sponge for miR-135a-3p, thereby upregulating STAT6, a key driver of M2 macrophage differentiation. Inhibition of LINC00313 or blockade of exosome release reduces M2 differentiation and tumor growth *in vivo*, establishing its role as a key mediator of tumor-macrophage communication (100).

**PCAT6** (Prostate Cancer-Associated Transcript 6) is significantly elevated in NSCLC tumor tissues and promotes tumor progression and immune modulation (101, 102). It acts as a ceRNA for miR-330-5p, promoting tumor proliferation and invasiveness (101). It also acts as a ceRNA for miR-326, thereby upregulating KLF1, a transcription factor that facilitates M2 macrophage polarization. PCAT6-loaded exosomes secreted by NSCLC cells are internalized by macrophages, reprogramming them towards an immunosuppressive M2 phenotype characterized by high IL-10 and low IL-1β expression (102).

**HOXC-AS2 (HOXC cluster antisense RNA 2)** is significantly upregulated in NSCLC tumor tissues and promotes tumor cell proliferation, migration, and EMT through interaction with HOXC13, a homeobox transcription factor (103). HOXC-AS2 and HOXC13 positively regulate each other, and HOXC13 silencing counteracts the pro-tumorigenic effects of HOXC-AS2 overexpression (103). Beyond its tumor cell-intrinsic role, HOXC-AS2 also functions as a key mediator in tumor–macrophage communication (104). It is selectively packaged into exosomes secreted by NSCLC cells and transferred to macrophages, where it induces M2 polarization. Mechanistically, cytoplasmic *HOXC-AS2* binds to STAT1, suppressing its activation and downstream SOCS1/CIITA signaling pathways essential for M1 polarization. Silencing *HOXC-AS2* in macrophages reverses this phenotype, limiting tumor proliferation and metastasis *in vivo* (104).

LINC01116 represents another extracellular vesicle (EV)-mediated mechanism by which NSCLC tumor cells reshape the immune microenvironment (105). Its expression is upregulated in both NSCLC tumor tissues and patient-derived circulating EVs, and high expression is associated with poor prognosis (105). Mechanistically, EV-transferred LINC01116 functions as a ceRNA by sponging miR-3614-5p, thereby upregulating ARHGAP1, a Rho GTPase-activating protein implicated in cytoskeletal remodeling. Notably, this axis operates in both tumor cells and macrophages — promoting proliferation, migration, and invasion in the former, and inducing M2 polarization characterized by elevated arginase-1, CD206, and IL-10 in the latter. Single-cell RNA sequencing confirmed LINC01116 expression across both epithelial and macrophage compartments, consistent with its dual functional role (105). These findings position LINC01116 as a potential prognostic biomarker and therapeutic target in NSCLC.

SNHG16 (Small nucleolar RNA host gene 16) is a hypoxia-driven EV-mediated mechanism of macrophage reprogramming in NSCLC (106). SNHG16 is significantly upregulated in tumor tissues compared to normal lung, and high expression correlates with poor prognosis, larger tumor size, metastasis, and advanced TNM stage (106). SNHG16 is also enriched in circulating EVs from NSCLC patients compared to healthy controls. Mechanistically, hypoxia-stimulated NSCLC cells secrete SNHG16-loaded EVs that are internalized by macrophages, inducing M2 polarization via a ceRNA mechanism — sponging miR-132-3p to upregulate KIF5A. M2-polarized macrophages in turn secrete IL-10, TGF-β, and VEGF, further enhancing tumor cell proliferation, EMT, and cancer stem cell properties, establishing a hypoxia-driven feedback loop between tumor cells and TAMs (106).

PURPL (p53 Upregulated Regulator of p53 Levels) functions as a tumor cell-intrinsic regulator of macrophage polarization (107). It is overexpressed in NSCLC tumor cells, as identified through bioinformatics analysis of the TCGA dataset, and its expression was validated in lung cancer cell lines. It facilitates macrophage polarization toward the M2 phenotype. PURPL binds to the RNA-binding protein RBM4, enhancing the stability of xCT (SLC7A11) mRNA, a key cystine/glutamate antiporter that protects cells from ferroptosis. Knockdown of xCT sensitizes macrophages to ferroptosis and inhibits M2 macrophage polarization (107).

## 4 Synthesis and emerging themes

Despite the molecular diversity of individual lncRNA transcripts, a recurring pattern emerges from the literature reviewed here: multiple lncRNAs converge on the same downstream immune regulatory nodes, suggesting that NSCLC maintains immunosuppression through functionally redundant but mechanistically distinct strategies. PD-L1 upregulation represents the most prominent convergence point, with at least seven distinct lncRNAs driving its overexpression through diverse molecular routes (**Figure 4**). At the transcriptional level, FGD5-AS1 sponges miR-454-3p to derepress the transcription factor ZEB1, which binds the PD-L1 promoter and drives PD-L1 transcription (73). At the post-transcriptional level, SNHG12 and SChLAP1 stabilize PD-L1 mRNA through interactions with the RNA-binding proteins HuR and AUF1, respectively (68, 69), while XIST, MALAT1, LINC01140, and SOX2-OT act as ceRNAs that sponge PD-L1-targeting miRNAs (71, 76–78). At the post-translational level, SNHG12 additionally stabilizes USP8 mRNA via HuR, and elevated USP8 deubiquitinates PD-L1 to prevent its proteasomal degradation (68). Conversely, LINC02418 promotes PD-L1 protein degradation via TRIM21-mediated ubiquitin-proteasomal proteolysis (75), while NKX2-1-AS1 acts as a decoy that sequesters the transcriptional activator NKX2-1, preventing its binding to the PD-L1 promoter (74). Collectively, these observations underscore that the PD-L1 axis is subject to bidirectional, multi-level lncRNA regulation within the NSCLC TME. The cGAS-STING pathway represents a second convergent target. NEAT1 suppresses it through DNMT1-mediated epigenetic repression (81), while PCAT1 independently downregulates cGAS expression via SOX2 (84) — two mechanistically distinct routes converging on the same innate immune axis.

M2 macrophage polarization constitutes a third convergent output, driven by ceRNA mechanisms (LINC00313, PCAT6, LINC01116, SNHG16, GNAS-AS1) (44, 100, 102, 105, 106), direct protein binding (HOXC-AS2 via STAT1 suppression) (104), and metabolic reprogramming via ADPGK-AS1, which binds mitochondrial proteins and enhances TCA cycle activity (47) — illustrating that tumor cells deploy diverse upstream molecular routes to promote the same immunosuppressive macrophage phenotype. Finally, bidirectional EV-mediated lncRNA transfer between tumor cells and macrophages emerges as a distinct regulatory theme. Tumor cell-derived EVs transfer LINC00313, PCAT6, HOXC-AS2, LINC01116, and SNHG16 to macrophages to promote M2 polarization, while M2 macrophage-derived EVs reciprocally deliver NORAD (45) and AGAP2-AS1 (48) to tumor cells, enhancing proliferation and radioresistance respectively. While the lncRNAs reviewed here converge on shared downstream nodes, a strictly defined convergent miRNA hub — wherein multiple distinct lncRNAs independently sponge the same miRNA within the NSCLC immune context — has not been conclusively demonstrated, representing a gap that systematic ceRNA network analyses in patient-derived datasets are well-positioned to address.

We also highlight context-dependent behavior as a defining characteristic of lncRNAs. MALAT1 illustrates this most clearly: it is downregulated in PBMCs of NSCLC patients, where its loss promotes MDSC expansion (56), yet upregulated in tumor tissue, where it drives PD-L1 upregulation and M2 macrophage recruitment (78, 79). Despite operating through compartment-specific mechanisms, both axes converge on the same functional outcome — promoting an immunosuppressive TME. SNHG6 provides a second example: it operates through ceRNA-mediated mechanisms in NSCLC tumor cells to promote proliferation, migration, and invasion (95), yet employs a protein ubiquitination pathway to promote M-MDSC differentiation (96) — expanding the immunosuppressive MDSC pool within the TME. PCAT6 sponges miR-330-5p in tumor cells to drive proliferation and invasion, yet engages a distinct miRNA target — miR-326 — in macrophages to promote M2 polarization (101, 102). Similarly, HOXC-AS2 interacts with HOXC13 in tumor cells to drive EMT and proliferation, while in macrophages it binds STAT1 to suppress M1 polarization signaling (103, 104). XIST similarly demonstrates cell-type specificity, upregulating PD-L1 in tumor cells while independently promoting M2 macrophage polarization in TAMs (71, 72). Collectively, these examples underscore that lncRNAs do not operate through fixed molecular programs — their interactomes, mechanisms, and downstream outputs are shaped by the cellular context in which they are expressed. These observations carry important implications for therapeutic development. Because the same lncRNA can serve opposing or distinct functions across cellular compartments, systemic targeting strategies risk unintended consequences. Achieving therapeutic specificity will therefore require cell-type-selective delivery approaches, and mechanistic conclusions drawn from models using a single cell type should be interpreted with appropriate caution.

Finally, the lncRNAs reviewed here span a wide spectrum of translational maturity. Those supported by convergent evidence from patient-derived tissues, functional *in vitro* studies, and *in vivo* preclinical models represent higher-confidence candidates for biomarker development or therapeutic exploration, while many rest on narrower evidentiary foundations — bioinformatic identification without experimental validation (NPSR1-AS1 (90)) or purely murine models or *in vitro* studies (AK036396 (57), linc-EPHA6-1 (97), SOX2-OT (77)). This unevenness reflects the pace of an emerging field, where discovery has outpaced validation, and systematic prioritization will be essential to identify candidates most ready for clinical translation.

## 5 Clinical implications of lncRNAs

### 5.1 LncRNAs as diagnostic and prognostic biomarkers

LncRNAs have emerged as promising noninvasive biomarkers for cancer detection due to their remarkable stability in bodily fluids and tumor-specific expression patterns, which may facilitate early detection with high sensitivity and specificity (108, 109). In early-stage NSCLC, elevated MALAT1 expression is significantly associated with increased metastatic risk and poor prognosis, underscoring its value as a prognostic indicator (80, 110). Moreover, the detection of MALAT1 in serum-derived exosomes further supports its potential as a noninvasive tool for disease monitoring (111). Several circulating lncRNAs and multi-lncRNA panels have demonstrated robust diagnostic performance in research cohorts. For instance, a four-lncRNA plasma panel (RMRP, NEAT1, TUG1, and MALAT1) achieved an area under the ROC curve (AUC) of 0.86–0.89, reflecting diagnostic accuracy (112). Similarly, a three-lncRNA plasma panel (SPRY4-IT1, ANRIL, and NEAT1) showed an AUC of 0.876 (113). Individually, SPRY4-IT1, ANRIL, and NEAT1 are significantly elevated in the plasma of NSCLC patients and exhibit high detection stability, making them attractive candidates for blood-based early-stage NSCLC screening (113). Similarly, XIST and HIF1A-AS1 are significantly upregulated in the serum of NSCLC patients compared with healthy controls, with their serum levels declining following surgical resection (70). Notably, their combined assessment yields superior diagnostic accuracy compared to either marker alone, highlighting their potential as complementary biomarkers for NSCLC screening (70). Additional lncRNAs, such as SChLAP1 and LINC00313, are also elevated in patient serum and correlate with poor clinical outcomes, reinforcing their dual diagnostic and prognostic relevance (69, 99). Beyond diagnosis and prognosis, certain lncRNAs may serve as predictive biomarkers of radiotherapeutic response. For instance, CBR3-AS1 and AGAP2-AS1 are upregulated in radioresistant NSCLC tissues, suggesting their potential to predict radiotherapy sensitivity (48, 86). Despite these promising findings, lncRNA-based assays are not yet used in clinical practice for NSCLC screening.

### 5.2 LncRNA-mediated regulation of ICI response and resistance

Despite the clinical success of immune checkpoint inhibitors including antibodies targeting PD-1, PD-L1, and CTLA-4 — in NSCLC (13, 14), only a subset of patients achieves durable responses (15–17), underscoring the need to identify molecular determinants of ICI efficacy and resistance. LncRNAs have emerged as key regulators of the TIME, modulating immune checkpoint molecule expression, immune cell infiltration, and cytokine production — thereby influencing tumor response or resistance to ICI therapy (114). LncRNAs that upregulate PD-L1 expression — including SNHG12, XIST, FGD5-AS1, and MALAT1 — facilitate tumor immune escape and may contribute to primary resistance to checkpoint blockade by maintaining high baseline PD-L1 levels in the TME (68, 69, 71, 73, 78). In contrast, lncRNAs such as NKX2-1-AS1 and LINC02418 function as negative regulators of PD-L1 expression, potentially sensitizing tumors to checkpoint blockade (74, 75). LINC02418 promotes PD-L1 protein degradation through TRIM21-mediated ubiquitination, and its restoration enhances anti-PD-L1 therapeutic efficacy in preclinical *in vivo* models. In NSCLC patients, LINC02418 expression is inversely correlated with PD-L1 levels and positively correlated with CD8+ T cell infiltration, predicting favorable clinical outcomes — positioning it as both a candidate predictive biomarker of ICI response and a potential therapeutic target (75). Beyond direct PD-L1 regulation, lncRNAs contribute to ICI resistance through immune exclusion and T cell suppression. NEAT1, PCAT1, CBR3-AS1, and LINC00301 restrict T cell infiltration, rendering tumors immune-excluded and potentially limiting ICI efficacy (81, 84, 86, 88). NKILA has been shown to promote AICD of cytotoxic T cells within the TME, reducing the anti-tumor effector pool (66). LINC00261 is a CDK1-related lncRNA that is downregulated in LUAD and associated with better prognosis — low LINC00261 expression correlates with high CDK1 and elevated CXCL8/IL-8 levels, which were increased in immunotherapy-resistant patients and decreased in responders, suggesting that the LINC00261/CDK1/CXCL8 axis may contribute to immune resistance in LUAD (115). Collectively, these findings position lncRNAs as multilevel regulators of ICI efficacy in NSCLC, operating through immune checkpoint-dependent mechanisms as well as broader pathways of T cell exclusion, effector cell depletion, and TME remodeling.

Beyond their mechanistic roles, lncRNAs also hold promise as predictive biomarkers of ICI response (116–118). Li et al. identified three lncRNA-defined TME subtypes in NSCLC with distinct immune phenotypes and differential immunotherapy sensitivity — an immune-inflamed subtype with superior survival and higher predicted ICI sensitivity, an immune-escape subtype characterized by interferon-driven MHC upregulation, and an immune-desert subtype with minimal immune infiltration (116). This lncRNA-based classification showed additive predictive value alongside PD-L1 expression and tumor mutational burden, suggesting it may complement existing biomarkers in guiding immunotherapy decisions — though prospective clinical validation remains warranted (116). Complementing this, Sun et al. developed a seven-lncRNA tumor-infiltrating immune-related lncRNA signature (TILSig) that classified NSCLC patients into immune-hot and immune-cold groups (117). Immune-hot patients with low TILSig combined with low immune checkpoint gene expression showed the most favorable outcomes, suggesting that combining lncRNA-based immune profiling with checkpoint gene expression may enable more precise patient selection for ICI therapy than either biomarker alone (117). Wang et al. further showed that plasma-derived exosomal lncRNA profiles differed between nivolumab responders and non-responders in NSCLC, with lnc-ZFP3–3 and lnc-CENPH-1/2 upregulated in non-responders, though the small sample size (n= 7) necessitates large-scale validation (118). Ren et al. developed a TIME-related lncRNA signature (TRLS) in LUAD, demonstrating that patients with high TRLS harbored elevated PD-L1 expression and showed superior sensitivity to immunotherapy across nine immunotherapeutic cohorts (119). Collectively, these lncRNA-based predictive frameworks remain largely exploratory — prospective clinical validation is lacking, cohort sizes are small, and how lncRNA expression evolves under ICI selective pressure remains poorly understood. Addressing these gaps will be essential to translate lncRNA-based immune profiling into actionable clinical biomarkers.

### 5.3 Therapeutic targeting of lncRNAs: strategies and challenges

Several strategies have been developed to modulate lncRNA activity, including small-molecule inhibitors, antisense oligonucleotides (ASOs), RNA interference (RNAi), locked nucleic acid (LNA) gapmers, CRISPR/Cas9-based genome editing, aptamers, and lncRNA mimics (120, 121). Small-molecule inhibitors can disrupt the secondary or tertiary structure of lncRNAs or interfere with their interactions with proteins, DNA, or other RNAs, thereby impairing their oncogenic functions (121). ASOs are short, single-stranded nucleic acids that hybridize to complementary lncRNA sequences, leading to transcript degradation via RNase H–mediated cleavage or by sterically blocking functional domains. LNA gapmers consist of a central DNA segment flanked by LNA-modified nucleotides, which confer high binding affinity and resistance to nuclease degradation. This design enables efficient RNase H–dependent knockdown of lncRNAs, including those localized in the nucleus (122). RNAi is another widely used approach, employing small interfering RNAs (siRNAs) or short hairpin RNAs (shRNAs) to guide the RNA-induced silencing complex (RISC) to complementary lncRNA sequences, resulting in sequence-specific cleavage and degradation (123, 124). CRISPR/Cas9 enables precise disruption or transcriptional repression of lncRNA loci, facilitating functional characterization and therapeutic exploration (125). Large-scale CRISPR interference (CRISPRi) screens have revealed that most functional lncRNAs exhibit highly cell-type-specific roles, underscoring the importance of context in their targeting (126). Aptamers are single-stranded oligonucleotides that adopt specific three-dimensional conformations, enabling them to bind directly to target lncRNAs and block their interactions with DNA, RNA, or protein partners (120). However, they are a less widely used approach compared to ASOs, RNAi, or CRISPR. In contrast to inhibitory approaches, lncRNA mimics are designed to restore or augment the function of tumor-suppressive lncRNAs whose expression is downregulated in cancer. These synthetic constructs replicate the structure and activity of endogenous lncRNAs, thereby reinstating their regulatory roles in transcriptional control, chromatin remodeling, or immune modulation (127).

Significant hurdles impede the successful translation of lncRNA therapies into clinical practice. One of the foremost challenges is delivery systems for lncRNA therapies. Safe and efficient delivery methods are essential to achieve precise tissue targeting while minimizing toxicity. Nanoparticles and exosomes have shown potential as delivery vehicles (128, 129). Nonetheless, each comes with significant drawbacks. Nanoparticles may induce toxicity and adverse immune responses, while exosome-based strategies face limitations such as high production costs, scalability issues, and lack of standardized protocols, reducing their feasibility for large-scale clinical use (127, 130). Beyond vehicle design, RNA-based therapeutics in general face additional obstacles. These include nonspecific uptake by undesired cells and off-target effects due to sequence similarity. Delivery inefficiency can also result in low bioavailability and poor therapeutic efficacy. Moreover, the inherent instability of naked, chemically unmodified RNA renders it highly susceptible to degradation. Finally, tolerability issues remain a concern, as recognition of foreign RNA structures by innate immune sensors such as Toll-like receptors (TLRs) may trigger adverse immune reactions (127). Another major challenge is the lack of sequence conservation and incomplete understanding of lncRNA function across species. This poses a significant hurdle when designing animal models for translational studies. In addition, the mechanisms by which lncRNAs exert their regulatory effects remain only partially understood. Identifying their downstream targets is complicated by the lack of efficient tools to accurately predict functional motifs and secondary or tertiary structures. These knowledge gaps limit the ability to design effective therapeutic strategies and raise the risk of off-target or unintended effects, thereby contributing to safety concerns in clinical applications.

### 5.4 Emerging technological frontiers: single-cell, spatial, and multi-omics lncRNA profiling

Conventional bulk RNA sequencing and RT-PCR-based validation have been foundational in identifying lncRNAs and characterizing their dysregulation in NSCLC. However, these approaches average expression signals across heterogeneous cell populations, obscuring cell-type-specific roles within the TIME. Single-cell RNA sequencing (scRNA-seq) has begun to address this limitation by resolving immune cell heterogeneity at single-cell resolution. Luo et al. systematically analyzed full-length scRNA-seq data from over 20,000 T cell libraries across colorectal cancer (CRC), NSCLC, and hepatocellular carcinoma (HCC), identifying 9,433 lncRNA genes — nearly doubling the known T cell lncRNA catalog — of which 154 signature lncRNAs were specifically associated with effector, exhausted, and regulatory T cell states, suggesting lncRNAs’ participation in T cell functional regulation within the TIME (131). Complementing this, Ren et al. integrated scRNA-seq with bulk RNA sequencing to identify a TIME-related lncRNA signature in LUAD (discussed in Section 5.2), demonstrating robust performance in predicting OS (119). Notably, scRNA-seq studies have identified T cell exhaustion subclusters in NSCLC using protein-coding gene signatures — including pre-exhausted, terminally exhausted, and regulatory states (132, 133), yet lncRNA expression across these functionally distinct populations remains largely uncharacterized, representing a critical gap that future scRNA-seq studies are well-positioned to address.

Spatial transcriptomics further extends these insights by preserving tissue architecture that is lost during cellular dissociation in scRNA-seq workflows (134). Alongside advancing computational methodologies, it is increasingly revolutionizing our understanding of tumor microenvironment organization and therapy response in cancer research (135). A recent study integrating scRNA-seq with spatial transcriptomics in NSCLC patients undergoing neoadjuvant chemoimmunotherapy revealed that tertiary lymphoid structures (TLS)-associated SELENOP-macrophages and their spatial co-localization with antigen-presenting cancer-associated fibroblasts (CAFs) at tumor boundaries correlated with therapeutic sensitivity (136). Notably, cell-cell communication between macrophages, CAFs, and T cells was spatially enhanced in responders, underscoring that spatially defined immune interactions carry functional significance that dissociation-based approaches cannot resolve (136). Yet to our knowledge, no study has mapped lncRNA expression within spatially defined niches such as TLS or tumor-stroma boundaries in NSCLC. Additionally, current spatial transcriptomics platforms, which predominantly capture polyadenylated mRNA, are not optimally designed for lncRNA detection — representing a key technological gap that next-generation platforms must address (134).

Beyond scRNA-seq and spatial transcriptomics, complementary approaches — including single-cell ATAC-sequencing (scATAC-seq), CITE-seq, long-read RNA sequencing, and m6A epitranscriptomic profiling — will be essential to fully resolve lncRNA biology in the NSCLC TIME. Long-read sequencing will enable isoform-resolved characterization of frequently dysregulated lncRNAs, whose splice variants may possess distinct immune regulatory functions (137, 138). m6A profiling will illuminate how post-transcriptional modifications regulate lncRNA stability and ceRNA activity across immune cell types, potentially explaining the context-dependent behaviors (139). Systematic lncRNA–protein interactome characterization via RNA immunoprecipitation coupled to mass spectrometry in purified immune cell populations will further clarify the mechanistic basis of cell-type-specific lncRNA functions (140). Together, these approaches will enable systematic mapping of lncRNA expression across diverse cell types and spatially defined tumor microenvironment niches, providing the foundation to identify high-confidence candidates for clinical translation.

## 6 Limitations

Despite the substantial body of evidence reviewed here, several important limitations of both the current field and this review must be acknowledged. A recurring methodological limitation across the lncRNA literature is the predominance of ceRNA-based mechanistic frameworks. The majority of studies reviewed here have functionally characterized lncRNAs primarily through miRNA sponging assays, and while this approach has yielded important insights, its biological significance depends on stoichiometric conditions — specifically, whether the lncRNA is expressed at sufficient copy numbers relative to the available miRNA pool to meaningfully compete for target binding — that are rarely quantified in primary tumor tissue or verified *in vivo* (141). While a proportion of lncRNAs in the current literature have been shown to function through direct protein interactions, characterized through RNA pulldown or RNA immunoprecipitation approaches, these studies largely identify binding partners rather than resolve the structural basis of the interaction. The structural domains of lncRNAs, the specific binding motifs that determine protein partner selectivity, and the kinetic dynamics of lncRNA-protein complex formation remain largely uncharacterized — reflecting a field-wide gap in which functional annotation has substantially outpaced structural understanding. Furthermore, the mechanistic studies reviewed here were conducted predominantly in cell line systems *in vitro*, and the isoform-specific expression and cell-type-specific interactome of most lncRNAs in NSCLC remain incompletely characterized — representing an important caveat in interpreting and generalizing these findings.

As noted in the introduction, an underexplored yet crucial gap in the field concerns lncRNA expression and function within specific immune cell subsets of the NSCLC TME. To date, there is little to no characterization of lncRNAs specifically expressed or functionally active in CD4^+^ T cell subsets — particularly Tregs — DCs, or NK cells within the lung TIME, despite their well-established roles in shaping tumor progression and immunotherapy outcomes. Furthermore, many lncRNA expression patterns described in this review are derived from bulk tissue data, which cannot distinguish immune cell-intrinsic signals from those of tumor or stromal cells — a resolution gap that scRNA-seq is well-positioned to address, yet such studies remain scarce in NSCLC patient samples.

A further limitation concerns the translational evidence base for many lncRNAs reviewed here. Studies comparing lncRNA expression profiles between ICI responders and non-responders in patient cohorts are largely absent, representing a missed opportunity to validate the lncRNAs implicated in immune checkpoint regulation. More broadly, the field remains heavily reliant on bioinformatic analyses of public datasets and preclinical models, with independent validation in large patient cohorts lacking for the majority of candidates. Importantly, several lncRNAs whose immune-modulatory roles have been characterized in murine models or *in vitro* systems have not been confirmed in human tumor-infiltrating immune cells — a critical gap given the significant differences in lncRNA sequence conservation and immune architecture between species (141). Where translational claims are made in this review, they reflect the potential of these molecules as candidates for further investigation rather than established clinical utility.

Finally, this review itself carries inherent limitations. The scope is necessarily selective — given the rapid expansion of the lncRNA literature, coverage of every reported lncRNA in NSCLC immunity was not feasible, and prioritization was based on mechanistic depth, immune relevance, and evidence quality. Our focus was deliberately restricted to lncRNAs with characterized roles in immune regulatory pathways within the NSCLC TIME — lncRNAs primarily implicated in tumor cell-intrinsic processes were not included. Furthermore, this review is subject to the publication bias inherent to the field — negative results and null findings in lncRNA biology are rarely published, meaning the landscape presented here likely overrepresents positive functional associations.

## 7 Conclusion and future directions

In this review, we have synthesized current knowledge on immune-regulatory lncRNAs in the NSCLC TIME. We have described their expression, prognostic associations, functional effects, and underlying molecular mechanisms, organized by the cell type in which they act, and graded each by level of supporting evidence to distinguish high-confidence translational candidates from exploratory observations. Building on this foundation, we have highlighted emerging themes including convergent regulatory nodes, diagnostic and prognostic biomarkers, lncRNA-mediated ICI response and resistance, therapeutic targeting strategies, and technological frontiers reshaping how lncRNAs can be studied in the TIME.

Looking ahead, translating these insights into clinical practice remains an open challenge — no clinical trials have yet evaluated the therapeutic use of lncRNAs in cancer, either as monotherapies or in combination with existing treatments. Bridging this translational gap requires integrating mechanistic discovery with clinically focused strategies. Silencing PD-L1-upregulating lncRNAs via ASOs or LNA gapmers in combination with anti-PD-1/PD-L1 antibodies could lower baseline PD-L1 in the TME and help overcome immunotherapy resistance, whereas restoration of PD-L1-suppressive lncRNAs such as LINC02418 through mimics or gene therapy may enhance checkpoint blockade efficacy — a strategy supported by preclinical evidence that LINC02418 restoration sensitizes tumors to anti-PD-L1 therapy (75). Targeting radioresistance-associated lncRNAs (e.g., AGAP2-AS1, CBR3-AS1) alongside radiotherapy could promote immunogenic cell death while impairing tumor-intrinsic survival pathways (48, 86). Targeting lncRNAs that drive MDSC expansion or M2-like TAM polarization could promote anti-tumor responses in combination regimens, particularly given that these myeloid populations expand following chemotherapy or EGFR-TKI treatment and blunt therapeutic efficacy (142, 143). NKILA silencing in CAR-T cells prior to adoptive transfer offers a particularly tractable cell-type-specific approach: ex vivo genetic engineering via shRNA or CRISPR-based editing circumvents systemic delivery challenges while enabling precise modulation of T cell function (66).

Realizing this potential requires dissecting lncRNA biology across immune cell states. signaling T cells, for example, display distinct distributions depending on tissue context— circulating T cells are predominantly naïve or effector, normal adjacent tissues are enriched in effector T cells, whereas tumor tissues are enriched in exhausted CD8^+^ T cells and Tregs (132). These functional states are likely regulated, at least in part, by lncRNAs. Thus, a deeper understanding of lncRNA expression patterns across naïve, effector, and exhausted states of CD8^+^ and CD4^+^ T cells is essential to unravel their roles in tumor-immune dynamics. Single-cell and spatial transcriptomic approaches will be particularly valuable here, offering the resolution to capture cell-type-specific and context-dependent lncRNA expression within the intact TME.

LncRNAs also hold promise as the next generation of noninvasive biomarkers in NSCLC, with emerging evidence supporting their utility in diagnostic, prognostic, and predictive contexts. Recent advances in protein-based immune targets such as delta-like ligand 3 (DLL3) in refractory SCLC further underscore the growing momentum toward biomarker-driven immunotherapy across lung cancer subtypes (144). Integrating non-coding and protein biomarker modalities may ultimately yield more robust frameworks for patient stratification and guide rational combination immunotherapies, moving the field closer to personalized treatment in lung cancer. Translating these opportunities will require prospective studies comparing lncRNA profiles between ICI responders and non-responders, independent cohort validation, and functional confirmation in human tumor-infiltrating immune cells for candidates currently characterized primarily *in vitro* or in murine models. Ultimately, forward initiating clinical trials will be a critical step to assess the feasibility, efficacy, and safety of lncRNA-based therapies in humans, and regulatory frameworks must evolve to accommodate non-coding RNA therapeutics that do not always fit conventional drug categories. This will require close collaboration among researchers, clinicians, and regulatory authorities to streamline development pipelines and ensure the safe and effective translation of lncRNA discoveries into clinical practice.
